# Non-Equilibrium Quantum Brain Dynamics: Super-Radiance and Equilibration in 2 + 1 Dimensions

**DOI:** 10.3390/e21111066

**Published:** 2019-10-30

**Authors:** Akihiro Nishiyama, Shigenori Tanaka, Jack A. Tuszynski

**Affiliations:** 1 Graduate School of System Informatics, Kobe University, 1-1 Rokkodai, Nada-ku, Kobe 657-8501, Japan; 2 Department of Oncology, University of Alberta, Cross Cancer Institute, Edmonton, AB T6G 1Z2, Canada; 3 Department of Physics, University of Alberta, Edmonton, AB T6G 2J1, Canada; 4 DIMEAS, Corso Duca degli Abruzzi, 24, Politecnico di Torino, 10129 Turin, TO, Italy

**Keywords:** non-equilibrium quantum field theory, quantum brain dynamics, Kadanoff–Baym equation, entropy, super-radiance

## Abstract

We derive time evolution equations, namely the Schrödinger-like equations and the Klein–Gordon equations for coherent fields and the Kadanoff–Baym (KB) equations for quantum fluctuations, in quantum electrodynamics (QED) with electric dipoles in 2+1 dimensions. Next we introduce a kinetic entropy current based on the KB equations in the first order of the gradient expansion. We show the H-theorem for the leading-order self-energy in the coupling expansion (the Hartree–Fock approximation). We show conserved energy in the spatially homogeneous systems in the time evolution. We derive aspects of the super-radiance and the equilibration in our single Lagrangian. Our analysis can be applied to quantum brain dynamics, that is QED, with water electric dipoles. The total energy consumption to maintain super-radiant states in microtubules seems to be within the energy consumption to maintain the ordered systems in a brain.

## 1. Introduction

Numerous attempts to understand memory in a brain have been made over one hundred years starting at the end of 19th century. Nevertheless, the concrete mechanism of memory still remains an open question in conventional neuroscience [1,2,3]. Conventional neuroscience is based on classical mechanics with neurons connected by synapses. However, we still cannot answer how limited connections between neurons describe mass excitations in a brain in classical neuron doctrine.

Quantum field theory (QFT) of the brain or quantum brain dynamics (QBD), is one of the hypotheses expected to describe the mechanism of memory in the brain [4,5,6]. Experimentally, several properties of memory, namely the diversity, the long-term but imperfect stability and nonlocality (Memory is diffused and non-localized in several domains in a brain. It does not disappear due to the destruction in a particular local domain. The term ‘nonlocality’ does not indicate nonlocality in entanglement in quantum mechanics.), are suggested in [7,8,9]. The QBD can describe these properties by adopting infinitely physically or unitarily inequivalent vacua in QFT, distinguished from quantum mechanics which cannot describe unitarily inequivalence. Unitarily inequivalence represents the emergence of the diversity of phases and allows the possibility of spontaneous symmetry breaking (SSB) [10,11,12,13]. The vacua or the ground states appearing in SSB describe the stability of the states. Furthermore, the QFT can describe both microscopic degrees of freedom and macroscopic matter [10]. To describe stored information, we can adopt the macroscopic ordered states in QFT with SSB involving long-range correlation via Nambu–Goldstone (NG) quanta. In 1967, Ricciardi and Umezawa proposed a quantum field theoretical approach to describe memory in a brain [14]. They adopted the SSB with long-range correlations mediated by NG quanta in QFT. Stuart et al. developed QBD by assuming a brain as a mixed system of classical neurons and quantum degrees of freedom, namely corticons and exchange bosons [15,16]. The vacua appearing in SSB, the macroscopic order, are interpreted as the memory storage in QBD. The finite number of excitations of NG modes represents the memory retrieval. Around the same time, Fröhlich proposed the application of a theory of electric dipoles to the study of biological systems [17,18,19,20,21,22]. He suggested a theory of the emergence of a giant dipole in open systems with breakdown of rotational symmetry of dipoles where dipoles are aligned in the same direction (the ordered states with coherent wave propagation of dipole oscillation in the Fröhrich condensate). In 1976, Davydov and Kislukha studied a theory of solitary wave propagation in protein chains, called the Davydov soliton [23]. It is found that the theory by Fröhlich and that by Davydov represent static and dynamical properties in the nonlinear Schödinger equation with an equivalent quantum Hamiltonian, respectively [24]. In the 1980s, Del Giudice et al. applied a theory of water electric dipoles to biological systems [25,26,27,28]. In particular, the derivation of laser-like behavior is a suggestive study. In the 1990s, Jibu and Yasue gave a concrete picture of corticons and exchange bosons, namely water electric dipole fields and photon fields [4,29,30,31,32]. The QBD is nothing but quantum electrodynamics (QED) with water electric dipole fields. When electric dipoles are aligned in the same directions coherently, the polaritons, NG bosons in SSB of rotational symmetry, emerge. The dynamical order in the vacua in SSB is maintained by long-range correlation of the massless NG bosons. In QED, the NG bosons are absorbed by photons and then photons acquire mass due to the Higgs mechanism and can stay in coherent domains. The massive photons are called evanescent photons. The size of a coherent domain is in the order of 50 μm. Furthermore, two quantum mechanisms of information transfer and integration among coherent domains are suggested. The first one is to use the super-radiance and the self-induced transparency via microtubules connecting two coherent domains [31]. Super-radiance is the phenomenon indicating coherent photon emission with correlation among not only photons but also atoms (or dipoles) [33,34,35,36,37]. The atoms (or dipoles) cooperatively decay in a short time interval due to correlation; coherent photons with intensity proportional to the square of the number of atoms (or dipoles) are emitted. The pulse wave photons in super-radiance propagate through microtubules without decay. Then the self-induced transparency appears, since microtubules are perfectly transparent in the propagation. The second one is to use the quantum tunneling effect among coherent domains surrounded by incoherent domains [32]. The effect is essentially equivalent to the Josephson effect between two superconducting domains separated by a normal domain. Del Giudice et al. studied this effect in biological systems [28]. In 1995, Vitiello has shown that a huge memory capacity can be realized by regarding a brain as an open dissipative system and doubling the degrees of freedom with mathematical techniques in thermo-field dynamics [38]. In dissipative model of a brain, each memory state evolves in classical deterministic trajectory like a chaos [39]. The overlap among distinct memory states is zero at any time in the infinite volume limit. However, finite volume effects allow states to overlap one another, which might represent association of memories [6]. In 2003, exclusion zone (EZ) water was discovered experimentally [40]. The properties of EZ water correspond to those of coherent water [41].

However, we have never seen the dynamical memory formations based on QBD at the physiological temperature in the presence of thermal effects written by quantum fluctuations. Hence, there are still criticisms related with the decoherence phenomena (We should use the mass of polaritons in estimating the critical temperature of ordered states, not that of water molecules themselves.)in memory formations in QBD [42]. So, we need to derive time evolution equations of coherent fields and quantum fluctuations and show numerical simulations of memory formation processes in non-equilibrium situations to check whether or not memory in QBD is robust against thermal effects. Futhermore, in 2012 Craddock et al. suggested the mechanism of memory coding in microtubules with phosphorylation by Ca${}^{2+}$ calmodulin kinase II [43]. It will be an interesting topic to investigate how water electric dipoles and evanescent photons are affected by phosphorylated microtubules.

The aim of this paper is to derive time evolution equations, namely the Schrödinger-like equations for coherent dipole fields, the Klein–Gordon equations for coherent photon fields, the Kadanoff–Baym equations for quantum fluctuations [44,45,46], with the two-particle-irreducible effective action technique with Keldysh formalism [47,48,49,50,51]. We derive both the equilibration for quantum fluctuations and the super-radiance for background coherent fields from the single Lagrangian in quantum electrodynamics (QED) with electric dipole fields. We arrive at the Maxwell–Bloch equations for the super-radiance by starting with QED with electric dipole fields in 2+1 dimensions. When we consider electric fields in super-radiance, we only need two spatial dimensions, one axis for the amplitude and another axis for the propagation. Hence we have discussed the case in 2+1 dimensions in this paper. By using our equations for super-radiance in this paper, we can describe information transfer via microtubules. Then, microtubule-associated proteins can make an important contribution to information transfer with interconnections among microtubules. We also derive the Higgs mechanism and the tachyonic instability for coherent fields in the Klein–Gordon equation for coherent electric fields. In two energy level approximation for electric dipole fields, namely with the ground state and the first excited states, the Higgs mechanism appears in normal population in which the probability amplitude in the ground state is larger than that in the first excited states. The penetrating length in the Meissner effect due to the Higgs mechanism is 6.3 μm derived by using coefficients in 2+1 dimensions and the number density of liquid water molecules in 3+1 dimensions. On the other hand, the tachyonic instability appears in inverted population in which the probability amplitudes in the first excited states are larger than that in the ground state. Then the electric field increases exponentially while the system is in inverted population. The increase stops at times when normal population is realized. Our analysis also contains the dynamics of quantum fluctuations in non-equilibrium cases. We also derive the Kadanoff–Baym equations for quantum fluctuations with the leading-order self-energy in the coupling expansion. The Kadanoff–Baym equations describe the entropy producing dynamics during equilibration as shown in the proof of the H-theorem. Entropy production stops when the Bose–Einstein distribution is realized. By combining time evolution equations (the Klein–Gordon equations for coherent electric fields and the Schrödinger-like equations for coherent electric dipole fields) and the Kadanoff–Baym equations for quantum fluctuations, we can describe the dynamical behavior of dipoles with thermal effects written by quantum fluctuations. Our analysis will be applied to memory formation processes in QBD. In particular, by extending our method to the case in open systems (networks), we can also trace dynamical memory recalling processes with excitations of particles in coherent domains via quantum tunneling processes, which are described by the Kadanoff–Baym equations. We can perform the simulations of the dynamical recalling processes in QBD with our equations to understand our thinking processes.

This paper is organized as follows. In Section 2, we introduce the two-particle-irreducible effective action in the closed-time path contour to describe non-equilibrium phenomena and derive time evolution equations. In Section 3, we introduce a kinetic entropy current in the first order of the gradient expansion, and show the H-theorem in the leading-order approximation of the coupling expansion. In Section 4, we show the time evolution equations, the conserved total energy and the potential energy in spatially homogeneous systems in an isolated system. In Section 5, we derive the super-radiance by analyzing the time evolution equations for coherent fields. In Section 6, we discuss our results. In Section 7, we provide the concluding remarks. In the Appendix A, we show how quantum fluctuations appear as additional terms in the Klein–Gordon equations. In this paper, the labels i,j=1 and 2 represent *x* and *y* directions in space, the labels a,b,c,d=1,2 represent two contours in the closed-time path, the labels α=−1,1 represent the angular momentum of electric dipoles. The speed of light, the Planck constant divided by 2π and the Boltzmann constant are set to be 1 in this paper. We adopt the metric tensor $\eta^{\mu \nu }=\mathrm{diag}\left(1,-1,-1\right)$ with μ,ν=0,1,2.

## 2. The Two-Particle-Irreducible Effective Action and Time Evolution Equations

We begin with the following Lagrangian density to describe quantum electrodynamics (QED) with electric dipoles in 2+1 dimensions in the background field method [52,53,54,55],

(1) $$\begin{array}{lll} L[\Psi^{*}\left(x,\theta \right),\Psi \left(x,\theta \right),A\left(x\right),a\left(x\right)] & = & -\frac{1}{4}F^{\mu \nu }\left[A+a\right]F_{\mu \nu }\left[A+a\right]-\frac{{\left(\partial^{\mu }a_{\mu }\right)}^{2}}{2\alpha_{1}}\\ & & +\int_{0}^{2\pi }d\theta [\Psi^{*}i\frac{\partial }{\partial x^{0}}\Psi +\frac{1}{2m}\Psi^{*}\nabla_{i}^{2}\Psi \\ & & +\frac{1}{2I}\Psi^{*}\frac{\partial^{2}}{\partial \theta^{2}}\Psi -2ed_{e}\Psi^{*}u^{i}\Psi F^{0i}\left[A+a\right]], \end{array}$$

where *A* is the background coherent photon fields, *a* is the quantum fluctuations of photon fields, $F^{\mu \nu }\left[A\right]=\partial^{\mu }A^{\nu }-\partial^{\nu }A^{\mu }$ is the field strength, the $\alpha_{1}$ is a gauge fixing parameter, the *m* is the mass of a dipole, the *I* is the moment of inertia, $u^{i}=\left(\cos\theta ,\sin\theta \right)$ is the direction of dipoles and $2ed_{e}$ is the absolute value of dipole vector. The variable θ represents the degrees of freedom of rotation of dipoles in 2+1 dimensions. The dipole–photon interaction term $-2ed_{e}\Psi^{*}u^{i}\Psi F^{0i}\left[A+a\right]$ has the similar form to that in [27]. We shall expand the electric dipole fields Ψ and $\Psi^{*}$ by the angular momentum and consider only the ground state and the first excited states in energy-levels. Then we can write them as,

(2) $$\begin{array}{lll} \Psi (x,\theta ) & = & \frac{1}{\sqrt{2\pi }}\left(\psi_{0}\left(x\right)+\psi_{1}\left(x\right)e^{i\theta }+\psi_{-1}\left(x\right)e^{-i\theta }\right),\\ \Psi^{*}\left(x,\theta \right) & = & \frac{1}{\sqrt{2\pi }}\left(\psi_{0}^{*}\left(x\right)+\psi_{1}^{*}\left(x\right)e^{-i\theta }+\psi_{-1}^{*}\left(x\right)e^{i\theta }\right), \end{array}$$

in 2+1 dimensions. (In 3+1 dimensions, we might expand Ψ and $\Psi^{*}$ by spherical harmonics.) We can rewrite the terms in the above Lagrangian as,

(3) $$\begin{array}{lll} \int d\theta \Psi^{*}\left(x,\theta \right)i\frac{\partial }{\partial x^{0}}\Psi \left(x,\theta \right) & = & \psi_{0}^{*}i\frac{\partial }{\partial x^{0}}\psi_{0}+\psi_{1}^{*}i\frac{\partial }{\partial x^{0}}\psi_{1}+\psi_{-1}^{*}i\frac{\partial }{\partial x^{0}}\psi_{-1}, \end{array}$$

(4) $$\begin{array}{lll} \int d\theta \frac{1}{2m}\Psi^{*}\nabla_{i}^{2}\Psi & = & \frac{1}{2m}\left[\psi_{0}^{*}\nabla_{i}^{2}\psi_{0}+\psi_{1}^{*}\nabla_{i}^{2}\psi_{1}+\psi_{-1}^{*}\nabla_{i}^{2}\psi_{-1}\right], \end{array}$$

(5) $$\begin{array}{lll} \int d\theta \frac{1}{2I}\Psi^{*}\frac{\partial^{2}}{\partial \theta^{2}}\Psi & = & \frac{-1}{2I}\left[\psi_{1}^{*}\psi_{1}+\psi_{-1}^{*}\psi_{-1}\right]. \end{array}$$

We also write the dipole–photon interaction term with electric fields $F^{0i}=-E_{i}$ by,

(6) $$\begin{array}{lll} \int d\theta 2ed_{e}\Psi^{*}u^{i}\Psi E_{i} & = & ed_{e}\int d\theta \left[\left(E_{1}-iE_{2}\right)\Psi^{*}e^{i\theta }\Psi +\left(E_{1}+iE_{2}\right)\Psi^{*}e^{-i\theta }\Psi \right]\\ & = & ed_{e}\left[\left(E_{1}-iE_{2}\right)\left(\psi_{0}^{*}\psi_{-1}+\psi_{1}^{*}\psi_{0}\right)+\left(E_{1}+iE_{2}\right)\left(\psi_{0}^{*}\psi_{1}+\psi_{-1}^{*}\psi_{0}\right)\right], \end{array}$$

with the direction of dipoles $u^{i}=\left(\cos\theta ,\sin\theta \right)$.

**Figure 1:**
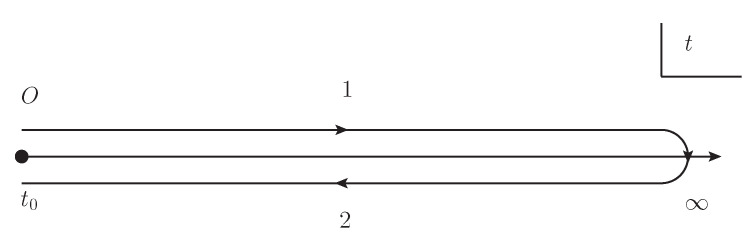
Closed-time path contour C. The label “1” represents the path from $t_{0}$ to *∞* and the label “2” represents the path from *∞* to $t_{0}$.

Next, we show two-particle-irreducible (2PI) effective action [47,48,49] for electric dipole fields and photon fields. Starting with the above Lagrangian density, we write the generating functional with the gauge fixing condition for quantum fluctuation,

(7) $$\begin{array}{l} \mathrm{gauge}\;\mathrm{fixing}\;:a^{0}=0, \end{array}$$

and perform the Legendre transformations. Then we arrive at,

(8) $$\begin{array}{lll} \Gamma_{2\mathrm{PI}}\left[A,{\bar{a}}^{i}\bar{\psi },{\bar{\psi }}^{*}\right] & = & \int_{C}d^{d+1}x[-\frac{1}{4}F^{\mu \nu }\left[A+\bar{a}\right]F_{\mu \nu }\left[A+\bar{a}\right]+i{\bar{\psi }}_{0}^{*}\frac{\partial }{\partial x_{0}}{\bar{\psi }}_{0}+\sum_{\alpha =-1,1}i{\bar{\psi }}_{\alpha }^{*}\frac{\partial }{\partial x_{0}}{\bar{\psi }}_{\alpha }\\ & & +\frac{1}{2m}\left({\bar{\psi }}_{0}^{*}\nabla_{i}^{2}{\bar{\psi }}_{0}+\sum_{\alpha =-1,1}{\bar{\psi }}_{\alpha }^{*}\nabla_{i}^{2}{\bar{\psi }}_{\alpha }\right)-\frac{1}{2I}\sum_{\alpha =-1,1}{\bar{\psi }}_{\alpha }^{*}{\bar{\psi }}_{\alpha }\\ & & +ed_{e}\sum_{\alpha =-1,1}\left[\left(E_{1}+i\alpha E_{2}\right)\left({\bar{\psi }}_{0}^{*}{\bar{\psi }}_{\alpha }+{\bar{\psi }}_{-\alpha }^{*}{\bar{\psi }}_{0}\right)\right]]\\ & & +i\mathrm{Tr}\ln\Delta^{-1}+i\mathrm{Tr}\Delta_{0}^{-1}\Delta +\frac{i}{2}\mathrm{Tr}\ln D^{-1}+\frac{i}{2}\mathrm{Tr}D_{0}^{-1}D+\frac{\Gamma_{2}\left[\Delta ,D\right]}{2}, \end{array}$$

where the C represents the Keldysh contour [50,51] shown in Figure 1, the spatial dimension d=2, the bar represents the expectation value 〈·〉 with the density matrix. The 3×3 matrix $i\Delta_{0}^{-1}\left(x,y\right)$ is defined as follows,

(9) $$\begin{array}{lll} i\Delta_{0}^{-1}\left(x,y\right) & \equiv & \frac{\delta^{2}\int_{x}L}{\delta \psi^{*}\left(y\right)\delta \psi \left(x\right)}{|}_{a=0}\\ & = & \left[\begin{array}{ccc} i\frac{\partial }{\partial x^{0}}+\frac{\nabla_{i}^{2}}{2m}-\frac{1}{2I} & ed_{e}\left(E_{1}+iE_{2}\right) & 0\\ ed_{e}\left(E_{1}-iE_{2}\right) & i\frac{\partial }{\partial x^{0}}+\frac{\nabla_{i}^{2}}{2m} & ed_{e}\left(E_{1}+iE_{2}\right)\\ 0 & ed_{e}\left(E_{1}-iE_{2}\right) & i\frac{\partial }{\partial x^{0}}+\frac{\nabla_{i}^{2}}{2m}-\frac{1}{2I} \end{array}\right]\delta_{C}^{d+1}\left(x-y\right), \end{array}$$

for −1, 0 and 1, and the $iD_{0,ij}^{-1}\left(x,y\right)$ is written by,

(10) $$\begin{array}{lll} iD_{0,ij}^{-1}\left(x,y\right) & \equiv & \frac{\delta^{2}\int_{x}L}{\delta a^{i}\left(x\right)\delta a^{j}\left(y\right)}\\ & = & -\delta_{ij}\partial_{x}^{2}\delta_{C}^{d+1}\left(x-y\right), \end{array}$$

where *i* and *j* run over spatial components 1,···,d=2 in 2+1 dimensions. The 3×3 matrix Δ(x,y) is,

(11) $$\begin{array}{lll} \Delta (x,y) & = & \left[\begin{array}{ccc} \Delta_{-1-1}\left(x,y\right) & \Delta_{-10}\left(x,y\right) & \Delta_{-11}\left(x,y\right)\\ \Delta_{0-1}\left(x,y\right) & \Delta_{00}\left(x,y\right) & \Delta_{01}\left(x,y\right)\\ \Delta_{1-1}\left(x,y\right) & \Delta_{10}\left(x,y\right) & \Delta_{11}\left(x,y\right) \end{array}\right], \end{array}$$

where $\Delta_{-10}\left(x,y\right)=\langle T_{C}\delta \psi_{-1}\left(x\right)\delta \psi_{0}^{*}\left(y\right)\rangle$ with time-ordered product $T_{C}$ in the closed-time path contour. The Green’s function of dipole fields $\Delta_{-10}\left(x,y\right)$ is also written by the 2×2 matrix $\Delta_{-10}^{ab}\left(x,y\right)$ with a,b=1,2 in the contour. The Green’s function for photon fields $D_{ij}\left(x,y\right)$ represents,

(12) $$\begin{array}{l} D_{ij}\left(x,y\right)=\langle T_{C}a_{i}\left(x\right)a_{j}\left(y\right)\rangle . \end{array}$$

Finally we write time evolution equations for coherent fields and quantum fluctuations. The 2PI effective action satisfies the following equations,

(13) $$\begin{array}{lll} \frac{\delta \Gamma_{2\mathrm{PI}}}{\delta \Delta }{|}_{\bar{a}=0} & = & 0, \end{array}$$

(14) $$\begin{array}{lll} \frac{\delta \Gamma_{2\mathrm{PI}}}{\delta D}{|}_{\bar{a}=0} & = & 0, \end{array}$$

(15) $$\begin{array}{lll} \frac{\delta \Gamma_{2\mathrm{PI}}}{\delta {\bar{a}}^{i}}{|}_{\bar{a}=0} & = & \frac{\delta \Gamma_{2\mathrm{PI}}}{\delta A^{i}}{|}_{\bar{a}=0}=0, \end{array}$$

(16) $$\begin{array}{lll} \frac{\delta \Gamma_{2\mathrm{PI}}}{\delta {\bar{\psi }}_{-1,0,1}^{\left(*\right)}}{|}_{\bar{a}=0} & = & 0, \end{array}$$

due to the Legendre transformation of the generating functional. Equation (13) is written by,

(17) $$\begin{array}{l} i\Delta_{0}^{-1}-i\Delta^{-1}-i\Sigma =0, \end{array}$$

with $i\Sigma \equiv -\frac{1}{2}\frac{\delta \Gamma_{2}}{\delta \Delta }$. The matrix of self-energy Σ can be written by diagonal elements,

(18) $$\begin{array}{l} \Sigma =\mathrm{diag}(\Sigma_{-1-1},\Sigma_{00},\Sigma_{11}), \end{array}$$

since we can neglect the off-diagonal elements which are higher order of the coupling expansion. Equation (17) represents the Kadanoff–Baym equations for electric dipole fields in the two-energy-level approximation in 2+1 dimensions. Similarly, the Kadanoff–Baym equation for photon fields in Equation (14) is written by,

(19) $$\begin{array}{l} iD_{0}^{-1}-iD^{-1}-i\Pi =0, \end{array}$$

with $i\Pi \equiv -\frac{\delta \Gamma_{2}}{\delta D}$. Equation (15) is given by,

(20) $$\begin{array}{l} \partial^{\nu }F_{\nu i}=J_{i}, \end{array}$$

with,

(21) $$\begin{array}{lll} J_{1}\left(x\right) & = & -ed_{e}\frac{\partial }{\partial x^{0}}\sum_{\alpha =-1,1}\left(\Delta_{0\alpha }\left(x,x\right)+\Delta_{\alpha 0}\left(x,x\right)+{\bar{\psi }}_{0}\left(x\right){\bar{\psi }}_{\alpha }^{*}\left(x\right)+{\bar{\psi }}_{\alpha }\left(x\right){\bar{\psi }}_{0}^{*}\left(x\right)\right), \end{array}$$

(22) $$\begin{array}{lll} J_{2}\left(x\right) & = & -ed_{e}\frac{\partial }{\partial x^{0}}\sum_{\alpha =-1,1}\left(-i\alpha (\Delta_{0\alpha }\left(x,x\right)-\Delta_{\alpha 0}\left(x,x\right)+{\bar{\psi }}_{0}\left(x\right){\bar{\psi }}_{\alpha }^{*}\left(x\right)-{\bar{\psi }}_{\alpha }\left(x\right){\bar{\psi }}_{0}^{*}\left(x\right))\right). \end{array}$$

Equation (20) represents the Klein–Gordon equations for spatial dimensions i=1 and 2. Equation (16) is written by,

(23) $$\begin{array}{lll} \left(i\frac{\partial }{\partial x^{0}}+\frac{\nabla_{i}^{2}}{2m}\right){\bar{\psi }}_{0}+\sum_{\alpha =-1,1}ed_{e}\left(E_{1}+i\alpha E_{2}\right){\bar{\psi }}_{\alpha } & = & 0, \end{array}$$

(24) $$\begin{array}{lll} \left(i\frac{\partial }{\partial x^{0}}+\frac{\nabla_{i}^{2}}{2m}-\frac{1}{2I}\right){\bar{\psi }}_{\alpha }+ed_{e}\left(E_{1}-i\alpha E_{2}\right){\bar{\psi }}_{0} & = & 0, \end{array}$$

and their complex conjugates. They are Schrödinger-like equations for coherent dipole fields. Equations (23) and (24) and their complex conjugates give the following probability conservation,

(25) $$\begin{array}{l} \frac{\partial }{\partial x^{0}}\left({\bar{\psi }}_{0}^{*}{\bar{\psi }}_{0}+\sum_{\alpha =-1,1}{\bar{\psi }}_{\alpha }^{*}{\bar{\psi }}_{\alpha }\right)+\frac{1}{2mi}\nabla_{i}\left({\bar{\psi }}_{0}^{*}\nabla_{i}{\bar{\psi }}_{0}-{\bar{\psi }}_{0}\nabla_{i}{\bar{\psi }}_{0}^{*}+\sum_{\alpha =-1,1}\left({\bar{\psi }}_{\alpha }^{*}\nabla_{i}{\bar{\psi }}_{\alpha }-{\bar{\psi }}_{\alpha }\nabla_{i}{\bar{\psi }}_{\alpha }^{*}\right)\right)=0. \end{array}$$

We shall define $J_{0}\left(x\right)$ as,

(26) $$\begin{array}{lll} J_{0}\left(x\right) & = & -ed_{e}\frac{\partial }{\partial x^{1}}\sum_{\alpha =-1,1}\left(\Delta_{0\alpha }\left(x,x\right)+\Delta_{\alpha 0}\left(x,x\right)+{\bar{\psi }}_{0}\left(x\right){\bar{\psi }}_{\alpha }^{*}\left(x\right)+{\bar{\psi }}_{\alpha }\left(x\right){\bar{\psi }}_{0}^{*}\left(x\right)\right)\\ & & -ed_{e}\frac{\partial }{\partial x^{2}}\left(-i\alpha (\Delta_{0\alpha }\left(x,x\right)-\Delta_{\alpha 0}\left(x,x\right)+{\bar{\psi }}_{0}\left(x\right){\bar{\psi }}_{\alpha }^{*}\left(x\right)-{\bar{\psi }}_{\alpha }\left(x\right){\bar{\psi }}_{0}^{*}\left(x\right))\right). \end{array}$$

Then since we can use $\partial_{0}J_{0}-\nabla_{i}J_{i}=0$ with i=1,2,

(27) $$\begin{array}{r} \partial_{0}J_{0}=\nabla_{i}J_{i}=-\partial^{i}\partial^{\nu }F_{\nu i}=\partial^{\mu }\partial^{\nu }F_{\nu \mu }-\partial^{i}\partial^{\nu }F_{\nu i}=\partial^{0}\partial^{\nu }F_{\nu 0},\\ \mathrm{or},\;\partial^{\nu }F_{\nu 0}=J_{0}, \end{array}$$

where the time dependent term in the time integral might be interpreted as an initial charge, but it is set to be zero. This equation represents the Poisson equation for scalar potential $A^{0}$ given by $\nabla^{2}A^{0}=\nabla \;\cdot \;\mu$ with the vector of dipole moments −μ on the right-hand side in Equation (26). (Since the Fourier transformed ${\tilde{A}}^{0}\left(q\right)$ is written by ${\tilde{A}}^{0}\left(q\right)\propto \left(q^{i}{\tilde{\mu }}_{i}\right)/q^{2}$ with $\mu_{i}={\tilde{\mu }}_{i}\delta \left(r\right)$, the electric field $E_{j}=-\nabla_{j}A^{0}\left(r\right)$ is proportional to $\int_{q}e^{iq\;\cdot \;r}\frac{q^{j}q^{i}{\tilde{\mu }}_{i}}{q^{2}}$. If we can also apply the analysis in this section to the case in 3+1 dimensions, we find $E_{j}\propto \partial_{j}\partial_{i}\frac{{\tilde{\mu }}_{i}}{r}$. Then we obtain dipole–dipole interaction potential $-{\tilde{\mu }}_{j}E_{j}∼\left[\frac{{\tilde{\mu }}_{j}{\tilde{\mu }}_{j}}{r^{3}}-\frac{3\left(r_{i}{\tilde{\mu }}_{i}\right)\left(r_{j}{\tilde{\mu }}_{j}\right)}{r^{5}}\right]$ in 3+1 dimensions.)

## 3. Kinetic Entropy Current in the Kadanoff–Baym Equations and the H-Theorem

In this section, we derive a kinetic entropy current from the Kadanoff–Baym equations with first order approximation of the gradient expansion and show the H-theorem for the leading-order approximations in the coupling expansion based on [56,57,58]. The analysis in this section is similar to that in open systems (the central region connected to the left and the right region) [59]. Since (−1,1) and (1,−1) components in $i\Delta_{0}^{-1}\left(x,y\right)$ in Equation (9) are zero, the same procedures to rewrite the Kadanoff–Baym equations as those in open systems [59,60,61,62,63] can be adopted. We set $t_{0}\to -\infty$.

First, we shall write the Kadanoff–Baym equations in Equation (17) for each components. By multiplying the matrix Δ from the right in Equation (17) and taking the (0,0) component, we can write it as,

(28) $$\begin{array}{l} i\left(\Delta_{0,00}^{-1}-\Sigma_{00}\right)\Delta_{00}+\sum_{\alpha =-1,1}ed_{e}\left(E_{1}+i\alpha E_{2}\right)\Delta_{\alpha 0}=i\delta_{C}\left(x-y\right), \end{array}$$

where the (0,0) component of the matrix $\Delta_{0}^{-1}$ represents $i\Delta_{0,00}^{-1}\left(x,y\right)=\left(i\frac{\partial }{\partial x^{0}}+\frac{\nabla_{i}^{2}}{2m}\right)\delta_{C}\left(x-y\right)$. By taking (α,0) component, we can write it as,

(29) $$\begin{array}{l} i\left(\Delta_{0,\alpha \alpha }^{-1}-\Sigma_{\alpha \alpha }\right)\Delta_{\alpha 0}+ed_{e}\left(E_{1}-i\alpha E_{2}\right)\Delta_{00}=0. \end{array}$$

It is convenient to introduce the Green’s functions $\Delta_{g,\alpha \alpha }$ as,

(30) $$\begin{array}{l} i\Delta_{g,\alpha \alpha }^{-1}=i\Delta_{0,\alpha \alpha }^{-1}-i\Sigma_{\alpha \alpha }. \end{array}$$

Then by using Equations (29) and (30), we can write $\Delta_{\alpha 0}$ as,

(31) $$\begin{array}{l} \Delta_{\alpha 0}\left(x,y\right)=-\frac{ed_{e}}{i}\int_{C}dw\Delta_{g,\alpha \alpha }\left(x,w\right)\left(E_{1}\left(w\right)-i\alpha E_{2}\left(w\right)\right)\Delta_{00}\left(w,y\right). \end{array}$$

Equation (31) means the propagation from *y* to *x* with zero angular momentum, change of angular momentum at *w* and the propagation from *w* to *x* with angular momentum α=±1. By using Equation (31), we can rewrite Equation (28) as,

(32) $$\begin{array}{r} i\int_{C}dw\left(\Delta_{0,00}^{-1}\left(x,w\right)-\Sigma_{00}\left(x,w\right)\right)\Delta_{00}\left(w,y\right)\\ +i\sum_{\alpha =-1,1}{\left(ed_{e}\right)}^{2}\int_{C}dw\left(E_{1}\left(x\right)+i\alpha E_{2}\left(x\right)\right)\Delta_{g,\alpha \alpha }\left(x,w\right)\left(E_{1}\left(w\right)-i\alpha E_{2}\left(w\right)\right)\Delta_{00}\left(w,y\right)=i\delta_{C}\left(x-y\right). \end{array}$$

The second term on the left-hand side in Equation (32) represents the propagation from *y* to *w* with zero angular momentum, the change of the angular momentum to α=±1 at *w* due to the coherent electric fields, the propagation from *w* to *x* and the change of the angular momentum from α=±1 to zero due to the coherent electric fields. In a similar way to $\phi^{4}$ theory in open systems [59], we can derive,

(33) $$\begin{array}{r} i\int_{C}dw\Delta_{00}\left(x,w\right)\left(\Delta_{0,00}^{-1}\left(w,y\right)-\Sigma_{00}\left(w,y\right)\right)\\ +i\sum_{\alpha =-1,1}{\left(ed_{e}\right)}^{2}\int_{C}dw\Delta_{00}\left(x,w\right)\left(E_{1}\left(w\right)+i\alpha E_{2}\left(w\right)\right)\Delta_{g,\alpha \alpha }\left(w,y\right)\left(E_{1}\left(y\right)-i\alpha E_{2}\left(y\right)\right)=i\delta_{C}\left(x-y\right), \end{array}$$

where we have used,

(34) $$\begin{array}{l} \Delta_{0\alpha }\left(x,y\right)=-\frac{1}{i}\int_{C}dw\Delta_{00}\left(x,w\right)\left(ed_{e}\right)\left(E_{1}\left(w\right)+i\alpha E_{2}\left(w\right)\right)\Delta_{g,\alpha \alpha }\left(w,y\right). \end{array}$$

The (α,α) components of the Kadanoff–Baym equations are written by,

(35) $$\begin{array}{r} i\int_{C}dw\left(\Delta_{0,\alpha \alpha }^{-1}\left(x,w\right)-\Sigma_{\alpha \alpha }\left(x,w\right)\right)\Delta_{\alpha \alpha }\left(w,y\right)\\ +i{\left(ed_{e}\right)}^{2}\int_{C}dw\left(E_{1}\left(x\right)-i\alpha E_{2}\left(x\right)\right)\Delta_{00}\left(x,w\right)\left(E_{1}\left(w\right)+i\alpha E_{2}\left(w\right)\right)\Delta_{g,\alpha \alpha }\left(w,y\right)=i\delta_{C}\left(x-y\right), \end{array}$$

and,

(36) $$\begin{array}{r} i\int_{C}dw\Delta_{\alpha \alpha }\left(x,w\right)\left(\Delta_{0,\alpha \alpha }^{-1}\left(w,y\right)-\Sigma_{\alpha \alpha }\left(w,y\right)\right)\\ +i{\left(ed_{e}\right)}^{2}\int_{C}dw\Delta_{g,\alpha \alpha }\left(x,w\right)\left(E_{1}\left(w\right)-i\alpha E_{2}\left(w\right)\right)\Delta_{00}\left(w,x\right)\left(E_{1}\left(x\right)+i\alpha E_{2}\left(x\right)\right)=i\delta_{C}\left(x-y\right), \end{array}$$

where we have used Equations (31) and (34).

Next, we shall perform the Fourier transformation ($\int d\left(x-y\right)e^{ip\;\cdot \;(x-y)}$) with the relative coordinate x−y of the (0,0) and (α,α) components of the Kadanoff–Baym equations. We use the 2×2 matrix notation in the closed-time path with a,b,c,d=1,2. Equations (32) and (33) are transformed as,

(37) $$\begin{array}{l} i{\left(\Delta_{0,00}^{-1}\left(p\right)-\Sigma_{00}\left(X,p\right)\sigma_{z}+\sum_{\alpha }U_{\alpha \alpha }\left(X,p\right)\sigma_{z}\right)}^{ac}\circ \Delta_{00}^{cb}\left(X,p\right)=i\sigma_{z}^{ab}, \end{array}$$

(38) $$\begin{array}{l} i\Delta_{00}^{ac}\left(X,p\right)\circ {\left(\Delta_{0,00}^{-1}\left(p\right)-\sigma_{z}\Sigma_{00}\left(X,p\right)+\sigma_{z}\sum_{\alpha }U_{\alpha \alpha }\left(X,p\right)\right)}^{cb}=i\sigma_{z}^{ab}, \end{array}$$

where $X=\frac{x+y}{2}$, $\sigma_{z}=\mathrm{diag}\left(1,-1\right)$,

(39) $$\begin{array}{l} i\Delta_{0,00}^{-1}\left(p\right)=p^{0}-\frac{p^{2}}{2m}, \end{array}$$

and the $U_{\alpha \alpha }\left(X,p\right)$ is the Fourier transformation,

(40) $$\begin{array}{lll} U_{\alpha \alpha }\left(X,p\right) & = & {\left(ed_{e}\right)}^{2}\int d\left(x-y\right)e^{ip\;\cdot \;(x-y)}\left(E_{1}\left(x\right)+i\alpha E_{2}\left(x\right)\right)\Delta_{g,\alpha \alpha }\left(x,y\right)\left(E_{1}\left(y\right)-i\alpha E_{2}\left(y\right)\right)\\ & = & {\left(ed_{e}\right)}^{2}E{\left(X\right)}^{2}\Delta_{g,\alpha \alpha }\left(X,p+\alpha \partial \zeta \right)+\left(\frac{\partial^{2}}{\partial X^{2}}\right), \end{array}$$

with the definition of ζ and |E|,

(41) $$\begin{array}{l} E_{1}\left(x\right)+i\alpha E_{2}\left(x\right)=\left|E\left(x\right)\right|e^{i\alpha \zeta (x)}, \end{array}$$

and,

(42) $$\begin{array}{l} {\left(U_{\alpha \alpha }\left(X,p\right)\sigma_{z}\right)}^{ac}=U_{\alpha \alpha }^{ad}\left(X,p\right)\sigma_{z}^{dc}, \end{array}$$

The ∘ is expanded by the derivative of *X* [64,65,66,67] as,

(43) $$\begin{array}{l} H\left(X,p\right)\circ I\left(X,p\right)=H\left(X,p\right)I\left(X,p\right)+\frac{i}{2}\left\{H,I\right\}+\left(\frac{\partial^{2}}{\partial X^{2}}\right), \end{array}$$

with the definition of the Poisson bracket,

(44) $$\begin{array}{l} \left\{H,I\right\}\equiv \frac{\partial H}{\partial p^{\mu }}\frac{\partial I}{\partial X_{\mu }}-\frac{\partial H}{\partial X^{\mu }}\frac{\partial I}{\partial p_{\mu }}. \end{array}$$

**Figure 2:**
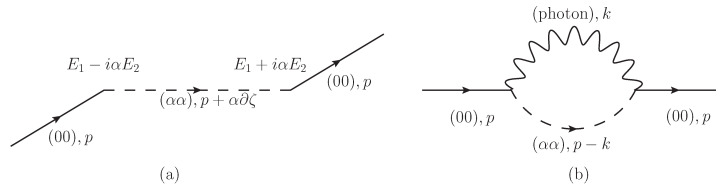
Diagrams of (**a**) $U_{\alpha \alpha }\left(X,p\right)$ and (**b**) self-energy $\Sigma_{00}\left(X,p\right)$.

We find that the $U_{\alpha \alpha }$ represents the change of momenta of dipoles as shown in Figure 2a.

In a similar way to [59], in the 0th and the first order in the gradient expansion in Equations (37) and (38), we can derive the following retarded Green’s function,

(45) $$\begin{array}{l} \Delta_{00,R}\left(X,p\right)=\frac{-1}{p^{0}-\frac{p^{2}}{2m}-\Sigma_{00,R}+\sum_{\alpha =-1,1}U_{\alpha \alpha ,R}}, \end{array}$$

with the retarded parts (the subscript ‘*R*’) $\Delta_{00,R}=i\left(\Delta_{00}^{11}-\Delta_{00}^{12}\right)$, $\Sigma_{00,R}=i\left(\Sigma_{00}^{11}-\Sigma_{00}^{12}\right)$ and $U_{\alpha \alpha ,R}=i\left(U_{\alpha \alpha }^{11}-U_{\alpha \alpha }^{12}\right)$. By taking the imaginary part of the retarded Green’s function $\Delta_{00,R}\left(X,p\right)$, we can derive the spectral function ρ00=i(Δ0021−Δ0012)=2iImΔ00,R(X,p) which represents the information of dispersion relations. Similarly, the (α,α) components of the Kadanoff–Baym equations are written as,

(46) $$\begin{array}{l} i\left(\Delta_{0,\alpha \alpha }^{-1}\left(p\right)-\Sigma_{\alpha \alpha }\left(X,p\right)\sigma_{z}\right)\circ \Delta_{\alpha \alpha }\left(X,p\right)+iV_{\alpha \alpha }\left(X,p\right)\sigma_{z}\circ \Delta_{g,\alpha \alpha }\left(X,p\right)=i\sigma_{z}, \end{array}$$

and,

(47) $$\begin{array}{l} i\Delta_{\alpha \alpha }\left(X,p\right)\circ \left(\Delta_{0,\alpha \alpha }^{-1}\left(p\right)-\sigma_{z}\Sigma_{\alpha \alpha }\left(X,p\right)\right)+i\Delta_{g,\alpha \alpha }\left(X,p\right)\circ \sigma_{z}V_{\alpha \alpha }\left(X,p\right)=i\sigma_{z}, \end{array}$$

where,

(48) $$\begin{array}{l} i\Delta_{0,\alpha \alpha }^{-1}\left(p\right)=p^{0}-\frac{p^{2}}{2m}-\frac{1}{2I}, \end{array}$$

and,

(49) $$\begin{array}{lll} V_{\alpha \alpha }\left(X,p\right) & = & {\left(ed_{e}\right)}^{2}\int d\left(x-y\right)e^{ip\;\cdot \;(x-y)}\left(E_{1}\left(x\right)-i\alpha E_{2}\left(x\right)\right)\Delta_{00}\left(x,y\right)\left(E_{1}\left(y\right)+i\alpha E_{2}\left(y\right)\right)\\ & = & {\left(ed_{e}\right)}^{2}E{\left(X\right)}^{2}\Delta_{00}\left(X,p-\alpha \partial \zeta \right)+\left(\frac{\partial^{2}}{\partial X^{2}}\right). \end{array}$$

We can also write for $\Delta_{g,\alpha \alpha }^{cb}\left(X,p\right)$ as,

(50) $$\begin{array}{lll} i{\left(\Delta_{0,\alpha \alpha }^{-1}\left(p\right)-\Sigma_{\alpha \alpha }\left(X,p\right)\sigma_{z}\right)}^{ac}\circ \Delta_{g,\alpha \alpha }^{cb}\left(X,p\right) & = & i\sigma_{z}^{ab}, \end{array}$$

(51) $$\begin{array}{lll} \Delta_{g,\alpha \alpha }^{ac}\left(X,p\right)\circ i{\left(\Delta_{0,\alpha \alpha }^{-1}\left(p\right)-\sigma_{z}\Sigma_{\alpha \alpha }\left(X,p\right)\right)}^{cb} & = & i\sigma_{z}^{ab}. \end{array}$$

In the 0th and the first order in the gradient expansion in Equations (46) and (47), we can derive,

(52) $$\begin{array}{l} \Delta_{\alpha \alpha ,R}=\Delta_{g,\alpha \alpha ,R}+\Delta_{g,\alpha \alpha ,R}V_{\alpha \alpha ,R}\Delta_{g,\alpha \alpha ,R} \end{array}$$

with $\Delta_{\alpha \alpha ,R}=i\left(\Delta_{\alpha \alpha }^{11}-\Delta_{\alpha \alpha }^{12}\right)$ and $V_{\alpha \alpha ,R}=i\left(V_{\alpha \alpha }^{11}-V_{\alpha \alpha }^{12}\right)$. Here we have used the solution in the 0th and the first order in the gradient expansion in Equations (50) and (51) given by,

(53) $$\begin{array}{l} \Delta_{g,\alpha \alpha ,R}=\frac{-1}{p^{0}-\frac{p^{2}}{2m}-\frac{1}{2I}-\Sigma_{\alpha \alpha ,R}}, \end{array}$$

with $\Sigma_{\alpha \alpha ,R}=i\left(\Sigma_{\alpha \alpha }^{11}-\Sigma_{\alpha \alpha }^{12}\right)$. The derivation is the same as [59]. The imaginary part of the retarded Green’s function $\Delta_{\alpha \alpha ,R}\left(X,p\right)$ multiplied by 2i represents the spectral function ραα=i(Δαα21−Δαα12)=2iImΔαα,R(X,p) which represents the information of dispersion relations. In addition, the Kadanoff–Baym equations for photons in Equation (19) are written by,

(54) $$\begin{array}{lll} i{\left(D_{0,ij}^{-1}\left(k\right)-\Pi_{ij}\left(X,k\right)\sigma_{z}\right)}^{ac}\circ D_{jl}^{cb}\left(X,k\right) & = & i\delta_{il}\sigma_{z}^{ab}, \end{array}$$

(55) $$\begin{array}{lll} iD_{ij}^{ac}\left(X,k\right)\circ {\left(D_{0,jl}^{-1}\left(k\right)-\sigma_{z}\Pi_{jl}\left(X,k\right)\right)}^{cb} & = & i\delta_{il}\sigma_{z}^{ab}, \end{array}$$

with,

(56) $$\begin{array}{l} iD_{0,ij}^{-1}\left(k\right)=k^{2}\delta_{ij}. \end{array}$$

Next we shall derive the self-energy in the leading-order (LO) of the coupling expansion in Equation (6). The (a,b)=(1,2) and (2,1) component of $i\frac{\Gamma_{2}}{2}$ are given by,

(57) $$\begin{array}{ccc} i\frac{\Gamma_{2,\mathrm{LO}}}{2} & = & -\frac{1}{2}{\left(ed_{e}\right)}^{2}\int dudw\sum_{\alpha =-1,1}(\Delta_{\alpha \alpha }^{21}\left(w,u\right)\Delta_{00}^{12}\left(u,w\right){\left(1,-\alpha i\right)}_{j}\partial_{u}^{0}\partial_{w}^{0}\left(D_{jl}^{12}\left(u,w\right)+D_{lj}^{21}\left(w,u\right)\right){\left(1,\alpha i\right)}_{l}^{t}\\ & & +\Delta_{\alpha \alpha }^{12}\left(w,u\right)\Delta_{00}^{21}\left(u,w\right){\left(1,-\alpha i\right)}_{j}\partial_{u}^{0}\partial_{w}^{0}\left(D_{jl}^{21}\left(u,w\right)+D_{lj}^{12}\left(w,u\right)\right){\left(1,\alpha i\right)}_{l}^{t}), \end{array}$$

where *t* represents the transposition. It is convenient to rewrite,

(58) $$\begin{array}{lll} D_{ij}^{ab}\left(k\right) & = & \left(\delta_{ij}-\frac{k_{i}k_{j}}{k^{2}}\right)D_{T}^{ab}\left(k\right)+\frac{k_{i}k_{j}}{k^{2}}D_{L}^{ab}\left(k\right), \end{array}$$

(59) $$\begin{array}{lll} \Pi_{ij}^{ab}\left(k\right) & = & \left(\delta_{ij}-\frac{k_{i}k_{j}}{k^{2}}\right)\Pi_{T}^{ab}\left(k\right)+\frac{k_{i}k_{j}}{k^{2}}\Pi_{L}^{ab}\left(k\right), \end{array}$$

where *T* and *L* represent the transverse and the longitudinal part, respectively. The LO self-energy $i\Pi_{ji}^{21}\left(y,x\right)=-\frac{\delta \Gamma_{2,\mathrm{LO}}}{\delta D_{ij}^{12}\left(x,y\right)}$ is,

(60) $$\begin{array}{lll} i\Pi_{jl}^{21}\left(y,x\right) & = & -i{\left(ed_{e}\right)}^{2}\sum_{\alpha =-1,1}(\partial_{x}^{0}\partial_{y}^{0}\left(\Delta_{\alpha \alpha }^{21}\left(y,x\right)\Delta_{00}^{12}\left(x,y\right)\right){\left(1,-\alpha i\right)}_{l}{\left(1,\alpha i\right)}_{j}^{t}\\ & & +\partial_{x}^{0}\partial_{y}^{0}\left(\Delta_{00}^{21}\left(y,x\right)\Delta_{\alpha \alpha }^{12}\left(x,y\right)\right){\left(1,-\alpha i\right)}_{j}{\left(1,\alpha i\right)}_{l}^{t}). \end{array}$$

By Fourier-transforming with the relative coordinate x−y and multiplying $\delta_{ij}-\frac{k_{i}k_{j}}{k^{2}}$ or $\frac{k_{i}k_{j}}{k^{2}}$, we arrive at,

(61) $$\begin{array}{lll} \Pi_{T}^{21}\left(X,k\right) & = & -{\left(ed_{e}\right)}^{2}{\left(k^{0}\right)}^{2}\int_{p}\sum_{\alpha =-1,1}\left(\Delta_{\alpha \alpha }^{21}\left(X,k+p\right)\Delta_{00}^{12}\left(X,p\right)+\Delta_{00}^{21}\left(X,k+p\right)\Delta_{\alpha \alpha }^{12}\left(X,p\right)\right)\\ & & +\left(\frac{\partial^{2}}{\partial X^{2}}\right), \end{array}$$

(62) $$\begin{array}{lll} \Pi_{L}^{21}\left(X,k\right) & = & \Pi_{T}^{21}\left(X,k\right), \end{array}$$

with $\int_{p}=\int \frac{d^{d+1}p}{{\left(2\pi \right)}^{d+1}}$. The second equation is due to the spatial dimension d=2. Similarly, we arrive at,

(63) $$\begin{array}{lll} \Pi_{T}^{12}\left(X,k\right) & = & -{\left(ed_{e}\right)}^{2}{\left(k^{0}\right)}^{2}\int_{p}\sum_{\alpha =-1,1}\left(\Delta_{\alpha \alpha }^{12}\left(X,k+p\right)\Delta_{00}^{21}\left(X,p\right)+\Delta_{00}^{12}\left(X,k+p\right)\Delta_{\alpha \alpha }^{21}\left(X,p\right)\right)\\ & & +\left(\frac{\partial^{2}}{\partial X^{2}}\right), \end{array}$$

(64) $$\begin{array}{lll} \Pi_{L}^{12}\left(X,k\right) & = & \Pi_{T}^{12}\left(X,k\right). \end{array}$$

The Fourier transformation of the LO self-energy $i\Sigma_{00}^{12}\left(x,y\right)=-\frac{1}{2}\frac{\delta \Gamma_{2,\mathrm{LO}}}{\delta \Delta_{00}^{21}\left(y,x\right)}$ is,

(65) $$\begin{array}{l} \Sigma_{00}^{12}\left(X,p\right)=-{\left(ed_{e}\right)}^{2}\int_{k}\sum_{\alpha =-1,1}{\left(k^{0}\right)}^{2}\Delta_{\alpha \alpha }^{12}\left(X,p-k\right)\left[D_{T}^{12}\left(X,k\right)+D_{L}^{12}\left(X,k\right)\right]+\left(\frac{\partial^{2}}{\partial X^{2}}\right). \end{array}$$

Similarly,

(66) $$\begin{array}{l} \Sigma_{00}^{21}\left(X,p\right)=-{\left(ed_{e}\right)}^{2}\int_{k}\sum_{\alpha =-1,1}{\left(k^{0}\right)}^{2}\Delta_{\alpha \alpha }^{21}\left(X,p-k\right)\left[D_{T}^{21}\left(X,k\right)+D_{L}^{21}\left(X,k\right)\right]+\left(\frac{\partial^{2}}{\partial X^{2}}\right). \end{array}$$

This self-energy is shown in Figure 2b. Similarly we can derive,

(67) $$\begin{array}{l} \Sigma_{\alpha \alpha }^{12}\left(X,p\right)=-{\left(ed_{e}\right)}^{2}\int_{k}{\left(k^{0}\right)}^{2}\Delta_{00}^{12}\left(X,p-k\right)\left[D_{T}^{12}\left(X,k\right)+D_{L}^{12}\left(X,k\right)\right]+\left(\frac{\partial^{2}}{\partial X^{2}}\right), \end{array}$$

and,

(68) $$\begin{array}{l} \Sigma_{\alpha \alpha }^{21}\left(X,p\right)=-{\left(ed_{e}\right)}^{2}\int_{k}{\left(k^{0}\right)}^{2}\Delta_{00}^{21}\left(X,p-k\right)\left[D_{T}^{21}\left(X,k\right)+D_{L}^{21}\left(X,k\right)\right]+\left(\frac{\partial^{2}}{\partial X^{2}}\right). \end{array}$$

Finally we derive a kinetic entropy current in the first order approximation in the gradient expansion and show the H-theorem in the LO approximation in the coupling expansion. By taking a difference of Equations (32) and (33), we arrive at,

(69) $$\begin{array}{l} i\left\{p^{0}-\frac{p^{2}}{2m},\Delta_{00}^{ab}\right\}=i{\left[\left(\Sigma_{00}-\sum_{\alpha }U_{\alpha \alpha }\right)\sigma_{z}\circ \Delta_{00}\right]}^{ab}-i{\left[\Delta_{00}\circ \sigma_{z}\left(\Sigma_{00}-\sum_{\alpha }U_{\alpha \alpha }\right)\right]}^{ab}. \end{array}$$

We use the Kadanoff–Baym Ansatz $\Delta_{00}^{12}=\frac{\rho_{00}}{i}f_{00}$, $\Delta_{00}^{21}=\frac{\rho_{00}}{i}\left(f_{00}+1\right)$, $\Sigma_{00}^{12}=\frac{\Sigma_{00,\rho }}{i}\gamma_{00}$, $\Sigma_{00}^{21}=\frac{\Sigma_{00,\rho }}{i}\left(\gamma_{00}+1\right)$, $U_{\alpha \alpha }^{12}=\frac{U_{\alpha \alpha ,\rho }}{i}\gamma_{U,\alpha \alpha }$ and $U_{\alpha \alpha }^{21}=\frac{U_{\alpha \alpha ,\rho }}{i}\left(\gamma_{U,\alpha \alpha }+1\right)$ with ρ00=i(Δ0021−Δ0012)=2iImΔ00,R, Σ00,ρ=i(Σ0021−Σ0012)=2iImΣ00,R and Uαα,ρ=i(Uαα21−Uαα12)=2iImUαα,R where we just rewrite the (1,2) and the (2,1) components with the spectral parts $\rho_{00}$, $\Sigma_{00,\rho }$ and $U_{\alpha \alpha ,\rho }$ and distribution functions $f_{00}$, $\gamma_{00}$ and $\gamma_{U,\alpha \alpha }$. The distribution functions $f_{00}$, $\gamma_{00}$ and $\gamma_{U,\alpha \alpha }$ approach the Bose–Einstein distributions near equilibrium states. In the first order approximation in the gradient expansion in Equation (69) for (a,b)=(1,2) and (2,1), we can derive,

(70) $$\begin{array}{l} f_{00}=\gamma_{00}+O\left(\frac{\partial }{\partial X}\right),\;\;\;\mathrm{and}\;\;\;f_{00}=\gamma_{U,\alpha \alpha }+O\left(\frac{\partial }{\partial X}\right). \end{array}$$

(Rewrite (a,b)=(1,2) and (2,1) components in Equation (69), then we can show the collision terms $\Delta_{00}^{21}\Sigma_{00}^{12}-\Delta_{00}^{12}\Sigma_{00}^{21}\propto f_{00}-\gamma_{00}=O\left(\frac{\partial }{\partial X}\right)$ and $f_{00}-\gamma_{U,\alpha \alpha }=O\left(\frac{\partial }{\partial X}\right)$.) We shall multiply $\ln\frac{i\Delta_{00}^{12}}{\rho_{00}}$ in (a,b)=(1,2) component in Equation (69) and $\ln\frac{i\Delta_{00}^{21}}{\rho_{00}}$ in (2,1) component in Equation (69), take the difference of them and integrate with $\int_{p}$. By the use of Equation (70), we arrive at,

(71) $$\begin{array}{lll} \partial_{\mu }s_{\mathrm{matter},00}^{\mu } & = & -\int_{p}\left(\Sigma_{00}^{21}\left(X,p\right)\Delta_{00}^{12}\left(X,p\right)-\Sigma_{00}^{12}\left(X,p\right)\Delta_{00}^{21}\left(X,p\right)\right)\ln\frac{\Delta_{00}^{12}\left(X,p\right)}{\Delta_{00}^{21}\left(X,p\right)}\\ & & +\sum_{\alpha }\int_{p}\left(U_{\alpha \alpha }^{21}\left(X,p\right)\Delta_{00}^{12}\left(X,p\right)-U_{\alpha \alpha }^{12}\left(X,p\right)\Delta_{00}^{21}\left(X,p\right)\right)\ln\frac{\Delta_{00}^{12}\left(X,p\right)}{\Delta_{00}^{21}\left(X,p\right)}, \end{array}$$

with the definition of entropy current $s_{\mathrm{matter},00}^{\mu }$ for (0,0) component,

(72) smatter,00μ≡∫p[δ0μ+δiμpim−∂Re(Σ00,R−∑αUαα,R)∂pμρ00i+∂ReΔ00,R∂pμΣ00,ρ−∑αUαα,ρi]σ[f00],

(73) $$\begin{array}{lll} \sigma [f_{00}] & \equiv & \left(1+f_{00}\right)\ln\left(1+f_{00}\right)-f_{00}\ln f_{00}. \end{array}$$

We can derive the Boltzmann entropy $\int_{p}\left[(1+n)\ln(1+n)-n\ln n\right]$ with the number density n(X,p) in the quasi-particle limit ImUαα,R=ImΣ00,R→0 in the same way as in [58]. Similarly, we can derive a kinetic entropy current for (αα) components. >From Equations (46) and (47), we can derive

(74) $$\begin{array}{lll} i\left\{p^{0}-\frac{p^{2}}{2m}-\frac{1}{2I},\Delta_{\alpha \alpha }^{ab}\right\} & = & i{\left[\Sigma_{\alpha \alpha }\sigma_{z}\circ \Delta_{\alpha \alpha }-\Delta_{\alpha \alpha }\circ \sigma_{z}\Sigma_{\alpha \alpha }\right]}^{ab}\\ & & -i{\left[V_{\alpha \alpha }\sigma_{z}\circ \Delta_{g,\alpha \alpha }-\Delta_{g,\alpha \alpha }\circ \sigma_{z}V_{\alpha \alpha }\right]}^{ab}. \end{array}$$

We use the Kadanoff–Baym Ansatz $\Delta_{\alpha \alpha }^{12}=\frac{\rho_{\alpha \alpha }}{i}f_{\alpha \alpha }$, $\Delta_{\alpha \alpha }^{21}=\frac{\rho_{\alpha \alpha }}{i}\left(f_{\alpha \alpha }+1\right)$, $\Delta_{g,\alpha \alpha }^{12}=\frac{\Delta_{g,\alpha \alpha ,\rho }}{i}\gamma_{g,\alpha \alpha }$, $\Delta_{g,\alpha \alpha }^{21}=\frac{\Delta_{g,\alpha \alpha ,\rho }}{i}\left(\gamma_{g,\alpha \alpha }+1\right)$, $\Sigma_{\alpha \alpha }^{12}=\frac{\Sigma_{\alpha \alpha ,\rho }}{i}\gamma_{\alpha \alpha }$, $\Sigma_{\alpha \alpha }^{21}=\frac{\Sigma_{\alpha \alpha ,\rho }}{i}\left(\gamma_{\alpha \alpha }+1\right)$, $V_{\alpha \alpha }^{12}=\frac{V_{\alpha \alpha ,\rho }}{i}\gamma_{V,\alpha \alpha }$ and $V_{\alpha \alpha }^{21}=\frac{V_{\alpha \alpha ,\rho }}{i}\left(\gamma_{V,\alpha \alpha }+1\right)$ with ραα=i(Δαα21−Δαα12)=2iImΔαα,R, Σαα,ρ=i(Σαα21−Σαα12)=2iImΣαα,R and Vαα,ρ=i(Vαα21−Vαα12)=2iImVαα,R. In Equation (74), we can show,

(75) $$\begin{array}{l} f_{\alpha \alpha }∼\gamma_{\alpha \alpha },\;\;\;\gamma_{g,\alpha \alpha }∼\gamma_{V,\alpha \alpha }, \end{array}$$

for distribution functions $f_{\alpha \alpha }$, $\gamma_{\alpha \alpha }$ and $\gamma_{V,\alpha \alpha }$ by writing the (a,b)=(1,2) and (2,1) components in the Kadanoff–Baym equations (74). We can also show,

(76) $$\begin{array}{l} \gamma_{\alpha \alpha }∼\gamma_{g,\alpha \alpha }, \end{array}$$

from Equations (50) and (51). We shall multiply $\ln\frac{i\Delta_{\alpha \alpha }^{12}}{\rho_{\alpha \alpha }}$ in (a,b)=(1,2) component in Equation (74) and $\ln\frac{i\Delta_{\alpha \alpha }^{21}}{\rho_{\alpha \alpha }}$ in (2,1) component in Equation (74), take the difference of them and integrate with $\int_{p}$. By using Equations (75) and (76), we arrive at,

(77) $$\begin{array}{lll} \partial_{\mu }s_{\mathrm{matter},\alpha \alpha }^{\mu } & = & -\int_{p}\left(\Sigma_{\alpha \alpha }^{21}\left(X,p\right)\Delta_{\alpha \alpha }^{12}\left(X,p\right)-\Sigma_{\alpha \alpha }^{12}\left(X,p\right)\Delta_{\alpha \alpha }^{21}\left(X,p\right)\right)\ln\frac{\Delta_{\alpha \alpha }^{12}\left(X,p\right)}{\Delta_{\alpha \alpha }^{21}\left(X,p\right)}\\ & & +\int_{p}\left(V_{\alpha \alpha }^{21}\left(X,p\right)\Delta_{g,\alpha \alpha }^{12}\left(X,p\right)-V_{\alpha \alpha }^{12}\left(X,p\right)\Delta_{g,\alpha \alpha }^{21}\left(X,p\right)\right)\ln\frac{\Delta_{\alpha \alpha }^{12}\left(X,p\right)}{\Delta_{\alpha \alpha }^{21}\left(X,p\right)}, \end{array}$$

with the definitions of entropy current $s_{\mathrm{matter},\alpha \alpha }^{\mu }$ for (αα) components,

(78) smatter,ααμ≡∫p[δ0μ+δiμpim−∂ReΣαα,R∂pμρααi+∂ReΔαα,R∂pμΣαα,ρi+∂ReVαα,R∂pμΔg,αα,ρi−∂ReΔg,αα,R∂pμVαα,ρi]σ[fαα].

In this derivation, we have used the same way as that in open systems in [59]. We can also derive the following equations for the Kadanoff–Baym equations for photons with the Kadanoff–Baym Ansatz $D_{T}^{21}=\frac{\rho_{T}}{i}\left(1+f_{T}\right)$, $D_{T}^{12}=\frac{\rho_{T}}{i}f_{T}$, $D_{L}^{21}=\frac{\rho_{L}}{i}\left(1+f_{L}\right)$ and $D_{L}^{12}=\frac{\rho_{L}}{i}f_{L}$ with distribution functions $f_{T}$ and $f_{L}$ and spectral functions $\rho_{T}$ and $\rho_{L}$,

(79) $$\begin{array}{lll} \partial_{\mu }s_{\mathrm{photon}}^{\mu } & = & -\frac{1}{2}\int_{k}\left[\Pi_{T}^{21}\left(X,k\right)D_{T}^{12}\left(X,k\right)-\Pi_{T}^{12}\left(X,k\right)D_{T}^{21}\left(X,k\right)\right]\ln\frac{D_{T}^{12}\left(X,k\right)}{D_{T}^{21}\left(X,k\right)}\\ & & -\frac{1}{2}\int_{k}\left[\Pi_{L}^{21}\left(X,k\right)D_{L}^{12}\left(X,k\right)-\Pi_{L}^{12}\left(X,k\right)D_{L}^{21}\left(X,k\right)\right]\ln\frac{D_{L}^{12}\left(X,k\right)}{D_{L}^{21}\left(X,k\right)}, \end{array}$$

with the entropy current for photons,

(80) sphotonμ≡∫kkμ−12∂ReΠT,R∂kμDT,ρi+12∂ReDT,R∂kμΠT,ρiσ[fT]+∫kkμ−12∂ReΠL,R∂kμDL,ρi+12∂ReDL,R∂kμΠL,ρiσ[fL].

As a result, the total entropy current $s^{\mu }=s_{\mathrm{matter},00}^{\mu }+\sum_{\alpha }s_{\mathrm{matter},\alpha \alpha }^{\mu }+s_{\mathrm{photon}}^{\mu }$ satisfies,

(81) $$\begin{array}{lll} \partial_{\mu }s^{\mu } & = & {\left(ed_{e}\right)}^{2}\int_{p,k}{\left(k^{0}\right)}^{2}\sum_{\alpha }\left[\Delta_{\alpha \alpha }^{21}\left(p-k\right)\Delta_{00}^{12}\left(p\right)D_{T}^{21}\left(k\right)-\Delta_{\alpha \alpha }^{12}\left(p-k\right)\Delta_{00}^{21}\left(p\right)D_{T}^{12}\left(k\right)\right]\\ & & \times \ln\frac{\Delta_{\alpha \alpha }^{21}\left(p-k\right)\Delta_{00}^{12}\left(p\right)D_{T}^{21}\left(k\right)}{\Delta_{\alpha \alpha }^{12}\left(p-k\right)\Delta_{00}^{21}\left(p\right)D_{T}^{12}\left(k\right)}\\ & & +{\left(ed_{e}\right)}^{2}\int_{p,k}{\left(k^{0}\right)}^{2}\sum_{\alpha }\left[\Delta_{\alpha \alpha }^{21}\left(p-k\right)\Delta_{00}^{12}\left(p\right)D_{L}^{21}\left(k\right)-\Delta_{\alpha \alpha }^{12}\left(p-k\right)\Delta_{00}^{21}\left(p\right)D_{L}^{12}\left(k\right)\right]\\ & & \times \ln\frac{\Delta_{\alpha \alpha }^{21}\left(p-k\right)\Delta_{00}^{12}\left(p\right)D_{L}^{21}\left(k\right)}{\Delta_{\alpha \alpha }^{12}\left(p-k\right)\Delta_{00}^{21}\left(p\right)D_{L}^{12}\left(k\right)}\\ & & +{\left(ed_{e}\right)}^{2}{\left(E\left(X\right)\right)}^{2}\sum_{\alpha }\int_{p}\left(\Delta_{g,\alpha \alpha }^{21}\left(p+\alpha \partial \zeta \right)\Delta_{00}^{12}\left(p\right)-\Delta_{g,\alpha \alpha }^{12}\left(p+\alpha \partial \zeta \right)\Delta_{00}^{21}\right)\\ & & \times \ln\frac{\Delta_{g,\alpha \alpha }^{21}\left(p+\alpha \partial \zeta \right)\Delta_{00}^{12}\left(p\right)}{\Delta_{g,\alpha \alpha }^{12}\left(p+\alpha \partial \zeta \right)\Delta_{00}^{21}\left(p\right)}\geq 0, \end{array}$$

where we have used the inequality $\left(x-y\right)\ln\frac{x}{y}\geq 0$ for real variables *x* and *y* with x>0 and y>0. The equality is satisfied in $f_{00}=f_{\alpha \alpha }=f_{T}=f_{L}=\frac{1}{e^{p^{0}/T}-1}$. Here we have used $\frac{\Delta_{\alpha \alpha }^{21}}{\Delta_{\alpha \alpha }^{12}}∼\frac{\Delta_{g,\alpha \alpha }^{21}}{\Delta_{g,\alpha \alpha }^{12}}$ with $\gamma_{g,\alpha \alpha }∼f_{\alpha \alpha }$ in first order in the gradient expansion. We have shown the H-theorem in the LO approximation in the coupling expansion and in the first order approximation in the gradient expansion. There is no violation in the second law in thermodynamics in the dynamics.

## 4. Time Evolution Equations in Spatially Homogeneous Systems and Conserved Energy

In this section, we write time evolution equations in spatially homogeneous systems and show a concrete form of the conserved energy density.

It is convenient to introduce the statistical functions $F_{00}=\frac{\Delta_{00}^{21}+\Delta_{00}^{12}}{2}$, $F_{\alpha \alpha }=\frac{\Delta_{\alpha \alpha }^{21}+\Delta_{\alpha \alpha }^{12}}{2}$, $F_{T}=\frac{D_{T}^{21}+D_{T}^{12}}{2}$, $F_{L}=\frac{D_{L}^{21}+D_{L}^{12}}{2}$, which represent the information of how many particles are occupied in $\left(p^{0},p\right)$ (particle distributions) and statistical parts,$U_{\alpha \alpha ,F}=\frac{U_{\alpha \alpha }^{21}+U_{\alpha \alpha }^{12}}{2}$, $V_{\alpha \alpha ,F}=\frac{V_{\alpha \alpha }^{21}+V_{\alpha \alpha }^{12}}{2}$, $\Delta_{g,\alpha \alpha ,F}=\frac{\Delta_{g,\alpha \alpha }^{21}+\Delta_{g,\alpha \alpha }^{12}}{2}$, $\Sigma_{00,F}=\frac{\Sigma_{00}^{21}+\Sigma_{00}^{12}}{2}$, $\Sigma_{\alpha \alpha ,F}=\frac{\Sigma_{\alpha \alpha }^{21}+\Sigma_{\alpha \alpha }^{12}}{2}$, $\Pi_{T,F}=\frac{\Pi_{T}^{21}+\Pi_{T}^{12}}{2}$ and $\Pi_{L,F}=\frac{\Pi_{L}^{21}+\Pi_{L}^{12}}{2}$. The variables of these functions are $\left(X^{0},p^{0},p\right)$ with the center-of-mass coordinate $X^{0}=\frac{x^{0}+y^{0}}{2}$ and *p* given by the Fourier transformation with the relative coordinate x−y in variables (x,y) in Green’s functions and self-energy in Section 2. The statistical functions and parts are real at any time when we start with real statistical functions at initial time. The spectral functions are given by taking the difference of (2,1) and (1,2) components multiplied by *i*, namely $\rho_{00}=i\left(\Delta_{00}^{21}-\Delta_{00}^{12}\right)$. They represent the information of which states can be occupied by particles in $\left(p^{0},p\right)$ (dispersion relations). The spectral parts in self-energy are given by taking the difference of (2,1) and (1,2) components multiplied by *i* (and written by the subscript ρ), namely $\Delta_{g,\alpha \alpha ,\rho }=i\left(\Delta_{g,\alpha \alpha }^{21}-\Delta_{g,\alpha \alpha }^{12}\right)$, $\Sigma_{00,\rho }=i\left(\Sigma_{00}^{21}-\Sigma_{00}^{12}\right)$ and so on. The spectral functions and parts are pure imaginary at any time when we start with pure imaginary spectral functions at initial time. We can use the real statistical parts labeled by the subscripts *F* and the pure imaginary spectral parts labeled by the subscript ρ in self-energy in the time evolution. We use the subscript ‘*R*’, ‘*F*’ and ‘ρ’ to represent the retarded, statistical and spectral parts in self-energy, respectively.

The Kadanoff–Baym equation for the statistical and spectral functions are given by,

(82) p0−p22m−ReΣ00,R+∑α=−1,1ReUαα,R,F00+ReΔ00,R,Σ00,F−∑αUαα,F=1iF00Σ00,ρ−ρ00Σ00,F−1i∑αF00Uαα,ρ−ρ00Uαα,F,

(83) p0−p22m−ReΣ00,R+∑α=−1,1ReUαα,R,ρ00+ReΔ00,R,Σ00,ρ−∑αUαα,ρ=0,

(84) p0−p22m−12I−ReΣαα,R,Fαα+ReΔαα,R,Σαα,F+ReVαα,R,Δg,αα,F−ReΔg,αα,R,Vαα,F=1iFααΣαα,ρ−ρααΣαα,F−1iΔg,αα,FVαα,ρ−Δg,αα,ρVαα,F,

(85) p0−p22m−12I−ReΣαα,R,ραα+ReΔαα,R,Σαα,ρ+ReVαα,R,Δg,αα,ρ−ReΔg,αα,R,Vαα,ρ=0,

(86) p0−p22m−12I−ReΣαα,R,Δg,αα,F+ReΔg,αα,R,Σαα,F=1iΔg,αα,FΣαα,ρ−Δg,αα,ρΣαα,F,

(87) p0−p22m−12I−ReΣαα,R,Δg,αα,ρ+ReΔg,αα,R,Σαα,ρ=0,

(88) p2−ReΠR,T,FT+ReDR,T,ΠF,T=1iFTΠρ,T−ρTΠF,T,

(89) p2−ReΠR,T,ρT+ReDR,T,Πρ,T=0,

and longitudinal parts given by changing the label *T* to *L* in Equations (88) and (89). We can use Equation (69) in the previous section to derive Equations (82) and (83), for example.

We can write,

(90) $$\begin{array}{ll} U_{\alpha \alpha ,F}\left(X,p\right)={\left(ed_{e}\right)}^{2}E{\left(X\right)}^{2}\Delta_{g,\alpha \alpha ,F}\left(p+\alpha \partial \zeta \right), & U_{\alpha \alpha ,\rho }\left(X,p\right)={\left(ed_{e}\right)}^{2}E{\left(X\right)}^{2}\Delta_{g,\alpha \alpha ,\rho }\left(p+\alpha \partial \zeta \right), \end{array}$$

(91) $$\begin{array}{ll} V_{\alpha \alpha ,F}\left(X,p\right)={\left(ed_{e}\right)}^{2}E{\left(X\right)}^{2}F_{00}\left(p-\alpha \partial \zeta \right), & V_{\alpha \alpha ,\rho }\left(X,p\right)={\left(ed_{e}\right)}^{2}E{\left(X\right)}^{2}\rho_{00}\left(p-\alpha \partial \zeta \right). \end{array}$$

In case we start with initial condition $E_{2}\left(X^{0}=0\right)=0$, $\partial_{0}E_{2}\left(X^{0}=0\right)=0$ and symmetric Green’s functions for α→−α in spatially homogeneous systems, we can use ∂ζ=0 in the above equations at any time. We can write the self-energy as,

(92) $$\begin{array}{lll} \Sigma_{00,F}\left(p\right) & = & -{\left(ed_{e}\right)}^{2}\sum_{\alpha =-1,1}\int_{k}{\left(k^{0}\right)}^{2}\left[F_{\alpha \alpha }\left(p-k\right)\left(F_{T}\left(k\right)+F_{L}\left(k\right)\right)+\frac{1}{4}\frac{\rho_{\alpha \alpha }\left(p-k\right)}{i}\frac{\rho_{T}\left(k\right)+\rho_{L}\left(k\right)}{i}\right], \end{array}$$

(93) $$\begin{array}{lll} \Sigma_{00,\rho }\left(p\right) & = & -{\left(ed_{e}\right)}^{2}\sum_{\alpha =-1,1}\int_{k}{\left(k^{0}\right)}^{2}\left[F_{\alpha \alpha }\left(p-k\right)\left(\rho_{T}\left(k\right)+\rho_{L}\left(k\right)\right)+\rho_{\alpha \alpha }\left(p-k\right)\left(F_{T}\left(k\right)+F_{L}\left(k\right)\right)\right], \end{array}$$

(94) $$\begin{array}{lll} \Sigma_{\alpha \alpha ,F}\left(p\right) & = & -{\left(ed_{e}\right)}^{2}\int_{k}{\left(k^{0}\right)}^{2}\left[F_{00}\left(p-k\right)\left(F_{T}\left(k\right)+F_{L}\left(k\right)\right)+\frac{1}{4}\frac{\rho_{00}\left(p-k\right)}{i}\frac{\rho_{T}\left(k\right)+\rho_{L}\left(k\right)}{i}\right], \end{array}$$

(95) $$\begin{array}{lll} \Sigma_{\alpha \alpha ,\rho }\left(p\right) & = & -{\left(ed_{e}\right)}^{2}\int_{k}{\left(k^{0}\right)}^{2}\left[F_{00}\left(p-k\right)\left(\rho_{T}\left(k\right)+\rho_{L}\left(k\right)\right)+\rho_{00}\left(p-k\right)\left(F_{T}\left(k\right)+F_{L}\left(k\right)\right)\right], \end{array}$$

(96) $$\begin{array}{lll} \Pi_{T,F}\left(k\right)=\Pi_{L,F}\left(k\right) & = & -{\left(ed_{e}\right)}^{2}{\left(k^{0}\right)}^{2}\sum_{\alpha =-1,1}\int_{p}[F_{\alpha \alpha }\left(k+p\right)F_{00}\left(p\right)-\frac{1}{4}\frac{\rho_{\alpha \alpha }\left(k+p\right)}{i}\frac{\rho_{00}\left(p\right)}{i}\\ & & +F_{00}\left(k+p\right)F_{\alpha \alpha }\left(p\right)-\frac{1}{4}\frac{\rho_{00}\left(k+p\right)}{i}\frac{\rho_{\alpha \alpha }\left(p\right)}{i}], \end{array}$$

(97) $$\begin{array}{lll} \Pi_{T,\rho }\left(k\right)=\Pi_{L,\rho }\left(k\right) & = & -{\left(ed_{e}\right)}^{2}{\left(k^{0}\right)}^{2}\sum_{\alpha =-1,1}\int_{p}[\rho_{\alpha \alpha }\left(k+p\right)F_{00}\left(p\right)-F_{\alpha \alpha }\left(k+p\right)\rho_{00}\left(p\right)\\ & & +\rho_{00}\left(k+p\right)F_{\alpha \alpha }\left(p\right)-F_{00}\left(k+p\right)\rho_{\alpha \alpha }\left(p\right)], \end{array}$$

where we have omitted the label of the center-of-mass cordinate *X* in Green’s functions and self-energy. We find that the $\Pi_{T,F}\left(k\right)=\Pi_{L,F}\left(k\right)$ are symmetric ($\Pi_{T,F}\left(-k\right)=\Pi_{T,F}\left(k\right)$) under k→−k and that $\Pi_{T,\rho }=\Pi_{L,\rho }$ are anti-symmetric ($\Pi_{T,\rho }\left(-k\right)=-\Pi_{T,\rho }\left(k\right)$) under k→−k, for any Green’s functions for dipole fields. When we prepare initial conditions with symmetric $F_{T,L}$ and anti-symmetric $\rho_{T,L}$ for photons, we can derive symmetric $F_{T,L}$ and anti-symmetric $\rho_{T,L}$ at any time. In addition, since Π(k)’s are proportional to ${\left(k^{0}\right)}^{2}$, there is no mass gap for incoherent photons for the leading-order self-energy in the coupling expansion. The velocity of gapless modes of incoherent photons will decrease when we increase the density of dipoles in this theory.

Finally, we show the energy density $E_{\mathrm{tot}}$. In the spatially homogeneous system in the 2+1 dimensions, we can derive $\frac{\partial E_{\mathrm{tot}}}{\partial X^{0}}=0$ with the energy density given by,

(98) Etot≡12I∑α=−1,1ψ¯α*ψ¯α+12∂0Ai2+∫pp0F00+∑α=−1,1Fαα+12∫pp02(FT+FL)+2(ede)2E2∑α=−1,1∫pF00(p)ReΔg,αα,R(p+α∂ζ)+ReΔ00,R(p)Δg,αα,F(p+α∂ζ)−∫pReΣ00,RF00+ReΔ00,RΣ00,F−∑α=−1,1∫pReΣαα,RFαα+ReΔαα,RΣαα,F−12∫pReΠR,TFT+ReDR,TΠF,T+ReΠR,LFL+ReDR,LΠF,L,

where we have used the KB equations in this section, the Klein–Gordon Equation (20) and the Schödinger-like Equations (23) and (24) in Section 2. The first term represents the contribution of nonzero angular momenta for coherent dipole fields. The second term represents the contribution by electric fields $E_{i}=\partial_{0}A_{i}$. The third and the fourth terms represent the contribution by quantum fluctuations for dipoles and photons, respectively. When the temperature is nonzero T≠0 at equilibrium states and the spectral width in the spectral functions is small enough, statistical functions which are proportional to the Bose–Einstein distributions $\frac{1}{e^{p^{0}/T}-1}$ give temperature-dependent terms $mT^{2}$ for dipole fields and $\propto T^{3}$ for photon fields in 2+1 dimensions. The fifth term represents the potential energy in processes in Figure 2a. The sixth, seventh and eighth terms represent the potential energy in processes in Figure 2b. The coefficients in the sixth and seventh terms are not $\frac{1}{3}$ but 1. While the factor 1 might look like a contradiction with the preceding research in [68,69] which suggest that the factor $\frac{1}{3}$ appears in the interaction with 3-point-vertex, the factor 1 appears due to time derivative ${\left(\partial^{0}\right)}^{2}$ in self-energy for dipole fields and photon fields.

## 5. Dynamics of Coherent Fields

In this section, we show that our Lagrangian describes the super-radiance phenomena in time evolution equations of coherent fields. We shall assume that all the coherent fields are independent of $x^{1}$ (dependent on $x^{0}$ and $x^{2}$). We also assume the symmetry for α=−1 and α=1, namely ${\bar{\psi }}_{1}^{\left(*\right)}={\bar{\psi }}_{-1}^{\left(*\right)}$, $\Delta_{01}=\Delta_{0-1}$, and $\Delta_{10}=\Delta_{-10}$. We set initial conditions $E_{2}=0$ and $\partial_{0}E_{2}=0$ at $x^{0}=0$.

We define $Z\equiv 2|{\bar{\psi }}_{1}{|}^{2}-{\left|{\bar{\psi }}_{0}\right|}^{2}$. It is possible to derive the following equations from time evolution Equations (20), (23) and (24) with their complex conjugates for background coherent fields in Section 2.

(99) $$\begin{array}{lll} \partial_{0}Z & = & i4ed_{e}E_{1}\left({\bar{\psi }}_{1}^{*}{\bar{\psi }}_{0}-{\bar{\psi }}_{0}^{*}{\bar{\psi }}_{1}\right), \end{array}$$

(100) $$\begin{array}{lll} \partial_{0}\left({\bar{\psi }}_{1}^{*}{\bar{\psi }}_{0}\right) & = & \frac{i}{2I}{\bar{\psi }}_{1}^{*}\psi_{0}+ied_{e}E_{1}Z \end{array}$$

(101) $$\begin{array}{lll} \left[{\left(\partial_{0}\right)}^{2}-{\left(\partial_{2}\right)}^{2}\right]E_{1} & = & -2ed_{e}{\left(\partial_{0}\right)}^{2}\left[{\bar{\psi }}_{1}^{*}{\bar{\psi }}_{0}+{\bar{\psi }}_{0}^{*}{\bar{\psi }}_{1}+\Delta_{01}\left(x,x\right)+\Delta_{10}\left(x,x\right)\right]. \end{array}$$

We have used moderately varying spatial dependence $|\nabla_{i}^{2}{\bar{\psi }}_{-1,0,1}/m|\ll |\partial_{0}{\bar{\psi }}_{-1,0,1}|$. We derive aspects of the super-radiance and the Higgs mechanism in the above three equations.

### 5.1. Super-Radiance

In this section, we show the super-radiance in time evolution equations for coherent fields with the rotating wave approximations neglecting non-resonant terms and quantum fluctuations. We have used the derivations in [70,71] for background coherent fields.

We shall consider only $k^{0}=\frac{1}{2I}$ in this section and we expand the electric field $E_{1}$ and the transition rate ${\bar{\psi }}_{0}{\bar{\psi }}_{1}^{*}$ as,

(102) $$\begin{array}{lll} E_{1}\left(x^{0},x^{2}\right) & = & \frac{1}{2}\epsilon \left(x^{0},x^{2}\right)e^{-i(k^{0}x^{0}-k^{0}x^{2})}+\frac{1}{2}\epsilon^{*}\left(x^{0},x^{2}\right)e^{i(k^{0}x^{0}-k^{0}x^{2})}, \end{array}$$

(103) $$\begin{array}{lll} {\bar{\psi }}_{1}{\bar{\psi }}_{0}^{*} & = & \frac{1}{2}R\left(x^{0},x^{2}\right)e^{-i(k^{0}x^{0}-k^{0}x^{2})}, \end{array}$$

We consider the following case,

(104) $$\begin{array}{ll} |\partial_{0}\epsilon |\ll |k^{0}\epsilon |, & |\partial_{0}R|\ll |k^{0}R|,\\ |\partial_{2}\epsilon |\ll |k^{0}\epsilon |. \end{array}$$

Neglect non-resonant terms like $e^{\pm 2ik^{0}x^{0}}$ and quantum fluctuations (Green’s functions $\Delta_{01}$ and $\Delta_{10}$) (the rotating wave approximation). Then from Equations (99)–(101), we arrive at the Maxwell–Bloch equations,

(105) $$\begin{array}{lll} \frac{\partial \epsilon }{\partial x^{0}}+\frac{\partial \epsilon }{\partial x^{2}} & = & ied_{e}k^{0}R, \end{array}$$

(106) $$\begin{array}{lll} \frac{\partial Z}{\partial x^{0}} & = & ied_{e}\left(\epsilon R^{*}-\epsilon^{*}R\right), \end{array}$$

(107) $$\begin{array}{lll} \frac{\partial R}{\partial x^{0}} & = & -ied_{e}\epsilon Z. \end{array}$$

We assume that ϵ, *Z* and *R* are independent of the spatial coordinate of the $x^{2}$ direction. We shall change ϵ→iϵ in the above equations and assume real functions $R=R^{*}$ and $\epsilon =\epsilon^{*}$. Then we can write,

(108) $$\begin{array}{lll} \frac{\partial \epsilon }{\partial x^{0}} & = & ed_{e}k^{0}R, \end{array}$$

(109) $$\begin{array}{lll} \frac{\partial Z}{\partial x^{0}} & = & -2ed_{e}\epsilon R, \end{array}$$

(110) $$\begin{array}{lll} \frac{\partial R}{\partial x^{0}} & = & ed_{e}\epsilon Z. \end{array}$$

We find the conservation law with the definition $B^{2}\equiv 2R^{2}+Z^{2}$,

(111) $$\begin{array}{l} \frac{\partial }{\partial x^{0}}B^{2}=\frac{\partial }{\partial x^{0}}\left(2R^{2}+Z^{2}\right)=0. \end{array}$$

The relation $\frac{\partial B}{\partial x^{0}}=0$ represents the probability conservation since we can rewrite $B^{2}={\left(2|{\bar{\psi }}_{1}{|}^{2}+{\left|{\bar{\psi }}_{0}\right|}^{2}\right)}^{2}$ by Equation (103) and $Z\equiv 2|{\bar{\psi }}_{1}{|}^{2}-{\left|{\bar{\psi }}_{0}\right|}^{2}$. We also find the following conservation law,

(112) $$\begin{array}{l} \frac{\partial }{\partial x^{0}}\left[\frac{1}{2}\epsilon^{2}+\frac{1}{2}k^{0}Z\right]=0, \end{array}$$

which represents the energy conservation. By this relation, we might be able to estimate the maximum energy density of electric fields,

(113) $$\begin{array}{l} {\left(\frac{1}{2}\epsilon^{2}\right)}_{\max}=-\frac{1}{2}k^{0}Z_{\min}=\frac{1}{2}k^{0}B, \end{array}$$

in case there is no external energy supply. We derive the following solutions in Equations (108)–(110),

(114) $$\begin{array}{l} R\left(x^{0}\right)=\frac{1}{\sqrt{2}}B\sin\theta \left(x^{0}\right),\;\;Z\left(x^{0}\right)=B\cos\theta \left(x^{0}\right), \end{array}$$

(115) $$\begin{array}{l} \theta \left(x^{0}\right)=\theta_{0}+\sqrt{2}ed_{e}\int_{0}^{x^{0}}d{x^{\prime }}^{0}\epsilon \left({x^{\prime }}^{0}\right), \end{array}$$

with $\frac{\partial \theta }{\partial x^{0}}=\sqrt{2}ed_{e}\epsilon$ and the constant *B* in a similar way to [71]. The $\theta (x^{0})$ swings around the position θ=π with the frequency $\Omega =ed_{e}\sqrt{k^{0}B}$ in case we start with initial conditions at around $\theta_{0}∼\pi$ ($|{\bar{\psi }}_{1}{|}^{2}=0$), since we can rewrite Equation (108) as

(116) $$\begin{array}{l} \frac{\partial^{2}\theta \left(x^{0}\right)}{\partial {\left(x^{0}\right)}^{2}}={\left(ed_{e}\right)}^{2}k^{0}B\sin\theta \left(x^{0}\right). \end{array}$$

The *B* is the order of the number density of dipoles.

We introduce the damping term $\frac{1}{L}\epsilon$ for the release of radiation and the propagation length *L* in Equation (108). We can write,

(117) $$\begin{array}{l} \frac{\partial \epsilon }{\partial x^{0}}+\frac{1}{L}\epsilon =\frac{ed_{e}k^{0}}{\sqrt{2}}B\sin\theta \left(x^{0}\right). \end{array}$$

In $\kappa =\frac{1}{L}\gg$ time derivative, we can neglect the first term in the above equations, then

(118) $$\begin{array}{l} \frac{\partial \theta }{\partial x^{0}}=\frac{{\left(ed_{e}\right)}^{2}k^{0}B}{\kappa }\sin\theta \left(x^{0}\right). \end{array}$$

The solution is,

(119) $$\begin{array}{l} \theta \left(x^{0}\right)=2\tan^{-1}\left[\exp\left(\frac{{\left(ed_{e}\right)}^{2}k^{0}Bx^{0}}{\kappa }\right)\tan\frac{\theta_{0}}{2}\right], \end{array}$$

and,

(120) $$\begin{array}{l} \epsilon =\frac{1}{\sqrt{2}ed_{e}\tau_{R}}\times {\left[\cosh\left(\frac{x^{0}-\tau_{0}}{\tau_{R}}\right)\right]}^{-1} \end{array}$$

with $\tau_{R}=\frac{\kappa }{{\left(ed_{e}\right)}^{2}k^{0}B}$ and $\tau_{0}=-\tau_{R}\ln\left(\tan\frac{\theta_{0}}{2}\right)$. The $\tau_{R}\propto 1/B∼$1/N with the number of dipoles *N* represents the relaxation time of electric fields in the super-radiance. When *N* dipoles decay within time scales 1/N, the intensity of electric fields becomes the order $N^{2}$ (super-radiant decay with correlation among dipoles), not *N* (spontaneous decay without correlation among dipoles).

### 5.2. Higgs Mechanism and Tachyonic Instability

In this section, we rewrite time evolution equations for coherent fields with only real functions. We assume the spatially homogeneous case. We do not adopt the rotating wave approximation in this section. We show how coherent electric fields $E_{1}$ are affected by $Z=2|{\bar{\psi }}_{1}{|}^{2}-{\left|{\bar{\psi }}_{0}\right|}^{2}$.

In Equation (101), the second derivatives of coherent fields on the right-hand side are written by,

$$\begin{array}{l} \frac{ed_{e}}{2I^{2}}\left({\bar{\psi }}_{1}^{*}\bar{\psi }+{\bar{\psi }}_{0}^{*}{\bar{\psi }}_{1}\right)+\frac{2{\left(ed_{e}\right)}^{2}Z}{I}E_{1}, \end{array}$$

where we have used Equation (100). As a result, we arrive at,

(121) (∂0)2−(∂2)2−2(ede)2ZIE1=μ14I2+2(ede)2E1I∫p(2F11(X,p)−F00(X,p)−Δg,11,F(X,p))+(ede)2I2E1∫pReΔg,11,R(X,p)F00(X,p)+Δg,11,F(X,p)ReΔ00,R(X,p)+(ede)22I2∂E1∂X0∫p∂F00∂p0Δg,11,ρi+ρ00i∂Δg,11,F∂p0+(ede)24I2E1∂∂X0∫p∂F00∂p0Δg,11,ρi+ρ00i∂Δg,11,F∂p0,

with the $x^{1}$ direction of the dipole moment (density) given by $\mu_{1}=2ed_{e}\left({\bar{\psi }}_{1}^{*}{\bar{\psi }}_{0}+{\bar{\psi }}_{0}^{*}{\bar{\psi }}_{1}\right)$, $F_{11}\left(X,p\right)=\frac{\Delta_{11}^{21}\left(X,p\right)+\Delta_{11}^{12}\left(X,p\right)}{2}$, $F_{00}\left(X,p\right)=\frac{\Delta_{00}^{21}\left(X,p\right)+\Delta_{00}^{12}\left(X,p\right)}{2}$ and $\Delta_{g,11,F}\left(X,p\right)=\frac{\Delta_{g,11}^{21}\left(X,p\right)+\Delta_{g,11}^{12}\left(X,p\right)}{2}$. In the Appendix A we have shown the detailed derivation for the second, third, fourth and fifth terms in the above equations. We have assumed the self-energy $\Sigma_{00}=\Sigma_{11}=0$ in deriving the time derivatives of $\Delta_{10}$ and $\Delta_{01}$ in Equation (101). Even if we include contributions of self-energy in Equation (121), they are higher order $O\left({\left(ed_{e}\right)}^{4}\right)$ in the coupling expansion. We have neglected higher order terms in the gradient expansion for quantum fluctuations. In Equation (121), we leave the $-{\left(\partial_{2}\right)}^{2}E_{1}$ term on the left-hand side in the above equation to compare with the sign of $-\frac{2{\left(ed_{e}\right)}^{2}Z}{I}E_{1}$ term. We find the Higgs mechanism with the mass squared $-\frac{2{\left(ed_{e}\right)}^{2}Z}{I}$ in the case of the normal population $Z=2|{\bar{\psi }}_{1}{|}^{2}-{\left|{\bar{\psi }}_{0}\right|}^{2}<0$. On the other hand, the tachyonic instability appears in the inverted population Z>0 in the above equation. Then the electric field $E_{1}$ will increase exponentially until *Z* becomes negative. In Equation (121), the second term on the right-hand side is proportional to $2F_{11}-F_{00}-\Delta_{g,11,F}$. Near equilibrium states, we might find $F_{00}>2F_{11}-\Delta_{g,11,F}$, where statistical functions $F_{11}$, $F_{00}$ and $\Delta_{g,11,F}$ are proportional to the Bose–Einstein distribution $\frac{1}{e^{p^{0}/T}-1}$ plus $\frac{1}{2}$ (with the Kadanoff–Baym ansatz) with different dispersion relations $p^{0}∼\frac{p^{2}}{2m}$ for $F_{00}$ and $p^{0}∼\frac{p^{2}}{2m}+\frac{1}{2I}$ for $F_{11}$ and $\Delta_{g,11,F}$, due to the energy difference $\frac{1}{2I}-\frac{0}{2I}$ between the ground state and first excited states. So the $2F_{11}-F_{00}-\Delta_{g,11,F}$ in the second term is negative near the equilibrium states, which might mean no tachyonic unstable terms appear from quantum fluctuations near equilibrium states. The contributions of quantum fluctuations on the right-hand side written by statistical functions (second, third, fourth and fifth terms) vanish at zero temperature T=0. Quantum fluctuations represent finite temperature effects at equilibrium states, although we need not restrict ourselves to only the equilibrium case. We have shown general contributions of quantum fluctuations in both equilibrium and non-equilibrium case in this paper.

Finally we shall consider remaining equations for coherent dipole fields. By using Equations (99) and (100) and the definitions of real functions $\mu_{1}=2ed_{e}\left({\bar{\psi }}_{1}^{*}{\bar{\psi }}_{0}+{\bar{\psi }}_{0}^{*}{\bar{\psi }}_{1}\right)$, $P=ied_{e}\left({\bar{\psi }}_{1}^{*}{\bar{\psi }}_{0}-{\bar{\psi }}_{0}^{*}{\bar{\psi }}_{1}\right)$ and $Z=2|{\bar{\psi }}_{1}{|}^{2}-{\left|{\bar{\psi }}_{0}\right|}^{2}$, we can also derive,

(122) $$\begin{array}{lll} \partial_{0}Z & = & 4E_{1}P, \end{array}$$

(123) $$\begin{array}{lll} \partial_{0}\mu_{1} & = & \frac{P}{I}, \end{array}$$

(124) $$\begin{array}{lll} \partial_{0}P & = & -\frac{\mu_{1}}{4I}-2{\left(ed_{e}\right)}^{2}E_{1}Z. \end{array}$$

We can show $\partial_{0}\left(2\right|{\bar{\psi }}_{1}{|}^{2}+|{\bar{\psi }}_{0}{|}^{2})=0$ by using these three equations. In these equations with initial conditions $E_{1}>0$, Z>0 (inverted population), P=0 and $\mu_{1}=0$, the *P* and the $\mu_{1}$ decrease at around the initial time and *Z* starts to decrease due to $E_{1}P<0$. In initial conditions $E_{1}>0$, Z<0 (normal population), P=0 and $\mu_{1}=0$, the *P* and the $\mu_{1}$ increase at around the initial time and *Z* starts to increase due to $E_{1}P>0$. The absolute values of *Z* decrease at around the initial time. We find that there is no term of quantum fluctuations in Equations (122)–(124).

We can solve Equations (121)–(124) with real functions in this section and the Kadanoff–Baym equations with real statistical functions and pure imaginary spectral functions in Section 4, simultaneously.

## 6. Discussion

In this paper, we have derived time evolution equations, namely the Klein–Gordon equations for coherent photon fields, the Schrödinger-like equations for coherent electric dipole fields and the Kadanoff–Baym equations for quantum fluctuations, starting with the Lagrangian in quantum electrodynamics with electric dipoles in 2+1 dimensions. We have adopted the two-particle-irreducible effective action technique with the leading-order self-energy of the coupling expansion. We find that electric dipoles change their angular momenta due to coherent electric fields $E_{1}\pm i\alpha E_{2}$ with α=±1. They also change momenta and angular momenta by scattering with incoherent photons. The proof of H-theorem is possible for these processes as shown in Section 3. Our analysis provides the dynamics of both the order parameters with coherent fields and quantum fluctuations for incoherent particles.

In Section 2, we adopt two-energy level approximation for the angular momenta of dipoles. Then, we find that the $i\Delta_{0}^{-1}$ is written by 3×3 matrix with zero (−1,1) and (1,−1) components. The form of the matrix is similar to 3×3 matrix in the analysis in open systems, the central region, left and right reservoirs as in [59,61,62,63]. Hence we can simplify the Kadanoff–Baym equations for dipole fields in an isolated system with the same procedures as those in open systems. The difference between QED with dipoles and $\phi^{4}$ theory in open systems is that the coherent electric field changes the momenta of dipoles when the phase αζ in $E_{1}\pm i\alpha E_{2}$ with α=±1 is dependent on space–time. The space dependence of coherent electric fields might disappear in the time evolution due to the instability by the lower entropy of the system, then electric fields will change angular momenta of dipoles but not change momenta *p* due to ∂ζ=0. We can also trace the dynamics with ∂ζ=0. By setting the initial conditions with the symmetry α→−α, namely ${\bar{\psi }}_{\alpha }^{\left(*\right)}={\bar{\psi }}_{-\alpha }^{\left(*\right)}$, $\Delta_{\alpha 0}=\Delta_{-\alpha 0}$ and $\Delta_{0\alpha }=\Delta_{0-\alpha }$, with initial conditions $E_{2}=0$ and $\partial_{0}E_{2}=0$ in spatially homogeneous systems in $\partial^{\nu }F_{\nu 2}=J_{2}$ in Equation (20), we can show $E_{2}=0$ at any time. Then we can use ∂ζ=0. This condition simplifies numerical simulations in the Kadanoff–Baym equations since we need not estimate the momentum shift p→p±α∂ζ in the finite-size lattice for the momentum space. As a result, the simulations for Kadanoff–Baym equations for dipoles and photons will be similar to those in QED with charged bosons in [72].

In Section 3, we have introduced a kinetic entropy current and shown the H-theorem in the leading-order of the coupling expansion with $ed_{e}$. This entropy approaches the Boltzmann entropy in the limit of zero spectral width as in [58]. The mode-coupling processes between dipoles and photons produce entropy. When there are deviations between (00) and (αα) components of distribution functions, entropy production occurs. Entropy production stops when the Bose–Einstein distribution is realized in the dynamics of Kadanoff–Baym equations.

We can also derive the energy shifts in dispersion relations due to nonzero electric fields by using the retarded Green’s functions in Section 3. The 0th order equations for retarded Green’s functions are given by,

(125) $$\begin{array}{lll} \left(p^{0}-\frac{p^{2}}{2m}+2{\left(ed_{e}\right)}^{2}E_{1}^{2}\Delta_{g,11,R}\right)\Delta_{00,R} & = & -1, \end{array}$$

(126) $$\begin{array}{lll} \left(p^{0}-\frac{p^{2}}{2m}-\frac{1}{2I}\right)\Delta_{11,R}+{\left(ed_{e}\right)}^{2}E_{1}^{2}\Delta_{00,R}\Delta_{g,11,R} & = & -1, \end{array}$$

with $\Delta_{g,11,R}=\frac{-1}{p^{0}-\frac{p^{2}}{2m}-\frac{1}{2I}}$. Multiply $p^{0}-\frac{p^{2}}{2m}-\frac{1}{2I}$, take the imaginary parts in the above equations and remember the imaginary parts of retarded Green’s functions are the spectral functions, then we find,

(127) $$\begin{array}{ccc} W\left[\begin{array}{c} \rho_{00}\\ \rho_{11} \end{array}\right] & = & 0,\;\mathrm{with},\\ W & = & \left[\begin{array}{cc} \left(p^{0}-\frac{p^{2}}{2m}-\frac{1}{2I}\right)\left(p^{0}-\frac{p^{2}}{2m}\right)-2{\left(ed_{e}\right)}^{2}E_{1}^{2} & 0\\ -{\left(ed_{e}\right)}^{2}E_{1}^{2} & {\left(p^{0}-\frac{p^{2}}{2m}-\frac{1}{2I}\right)}^{2} \end{array}\right] \end{array}$$

By setting determinant |W| to be zero, we find the following solutions for dispersion relations,

(128) $$\begin{array}{l} p^{0}=\frac{p^{2}}{2m}+\frac{1}{4I}\pm \frac{1}{2}\sqrt{\frac{1}{4I^{2}}+8{\left(ed_{e}\right)}^{2}{E_{1}}^{2}}. \end{array}$$

Here we assumed the symmetry for α=±1 for Green’s functions and zero self-energy $\Sigma_{00}=\Sigma_{11}=0$. We find how electric fields shift two energy levels 0 and $\frac{1}{2I}$. The above energy shift is similar to the energy shift given in [27] in 3+1 dimensions due to nonzero electric fields.

In Section 5.1, we have derived the super-radiance from time evolution equations for coherent fields. We find that it is possible to derive the Maxwell–Bloch equations from our Lagrangian with the probability conservation law and the energy conservation law. Super-radiant decay with intensity of the order $\propto N^{2}$ (*N*: The number of dipoles) appears in a similar way to [70,71]. It is possible to derive the maximum energy of electric fields by use of Equation (113). We know that the moment of inertia of water molecule is $I=2m_{H}R^{2}$ with $m_{H}=940$ with $R=0.96\times 10^{-10}$m. Hence the $k^{0}=\frac{1}{2I}=1.1\times 10^{-3}$eV. Since $B=\frac{N}{V}=3.3\times 10^{28}$$/m^{3}$ for liquid water, we find

(129) $$\begin{array}{l} \frac{1}{2}\epsilon_{\max}^{2}=\frac{1}{2}k^{0}B=1.8\times 10^{25}\;\mathrm{eV}/m^{3}. \end{array}$$

When we multiply the volume of all microtubules (MTs) in a brain,

(130) $$\begin{array}{lll} V_{\mathrm{MT}} & = & \pi \times {15\mathrm{nm}}^{2}\times 1000\mathrm{nm}\times 2000\;\mathrm{MTs}/\mathrm{neuron}\times 10^{11}\;\mathrm{neurons}/\mathrm{brain}=1.4\times 10^{-7}\;m^{3}, \end{array}$$

we can arrive at,

(131) $$\begin{array}{l} \frac{1}{2}\epsilon_{\max}^{2}V_{\mathrm{MT}}=0.41\;J=0.1\;\mathrm{cal}. \end{array}$$

If we maintain our brain 100s without energy supply, we need at least $0.1\times 10^{-2}\;\mathrm{cal}/s$ or 86cal/day to maintain the ordered states of memory. We can compare 86cal/day with 4000cal/day=2000kcal/day×0.2(energyconsumptionrateofbrain)×0.01(energyratetomaintaintheorderedsystem). The 86cal/day is within the 4000cal/day, which is consistent with our experiences. In this derivation, we have used coefficients in 2+1 dimensions and the number density of water molecules in 3+1 dimensions.

In Section 5.2, we have derived time evolution equations for electric field $E_{1}$. The Higgs mechanism appears in this equation in normal population Z<0. As a result, the dynamical mass generation occurs with the maximum mass $\Omega_{\mathrm{Higgs}}=2ed_{e}\sqrt{k^{0}B}=30k^{0}$ where the number density of dipoles is $B=2|{\bar{\psi }}_{1}{|}^{2}+{\left|{\bar{\psi }}_{0}\right|}^{2}=\frac{N}{V}$. The period is $2\pi /\Omega_{\mathrm{Higgs}}=1.3\times 10^{-13}\;s$. In normal population Z<0, the Meissner effect appears with the penetrating length $1/\Omega_{\mathrm{Higgs}}=6.3\;\mu m$. On the other hand, the tachyonic instability occurs in inverted population Z>0. The electric field $E_{1}$ increases exponentially with $\exp(\Omega X^{0})$ (with $\Omega \leq \Omega_{\max}$) where the time scale is $1/\Omega_{\max}=2.1\times 10^{-14}\;s$ with $\Omega_{\max}=\Omega_{\mathrm{Higgs}}$. Due to energy conservation, since *Z* decreases as the absolute value of the electric field increases, tachyonic instability stops in Z<0.

We have prepared for numerical simulations with time evolution equations, namely the Schödinger-like equations for coherent electric dipole fields, the Klein–Gordon equations for coherent electric fields and the Kadanoff–Baym equations for quantum fluctuations. Our simulations might describe the dynamics towards equilibrium states for quantum fluctuations and the dynamics of super-radiant states for coherent fields. Our analysis is also extended to simulations in open systems by preparing the left and the right reservoirs like those in [59] or networks [73].

## 7. Conclusions

We have derived the Schrödinger equations for coherent electric dipole fields, the Klein–Gordon equations for coherent electric fields and the Kadanoff–Baym equations for quantum fluctuations in QED with electric dipoles in 2+1 dimensions. It is possible to derive equilibration for quantum fluctuations and super-radiance for background coherent fields simultaneously. Total energy consumption to maintain super-radiance in microtubules is consistent with energy consumption in our experiences. We can describe dynamical information transfer with super-radiance via microtubules without violation of the second law in thermodynamics. We have also derived the Higgs mechanism in normal population and the tachyonic instability in inverted population. These dynamical properties might be significant to form and maintain coherent domains composed of dipoles and photons. We are ready to describe memory formation processes towards equilibrium states in 2+1 dimensions with equations in this paper. Furthermore, our approach might pave the way to understand the dynamical thinking processes with memory recalling in QBD by investigating the case in open systems with the Kadanoff–Baym equations. This work will be extended to the 3+1 dimensional analysis to describe memory formation processes in numerical simulations. We should derive the Schödinger-like equations, the Klein–Gordon equations and the Kadanoff–Baym equations by starting with the single Lagrangian in QED with electric dipoles in 3+1 dimensions in the future study. These equations in 3+1 dimensions will describe more realistic and practical dynamics in QBD.

## Appendix A. Quantum Fluctuations in the Klein–Gordon Equations

In this section, we shall derive the second, third, fourth and fifth terms involving quantum fluctuations on the right-hand side in Equation (121) in spatially homogeneous systems. They correspond to the following term,

$$\begin{array}{l} -2ed_{e}{\left(\partial_{0}\right)}^{2}\left[\Delta_{10}\left(x,x\right)+\Delta_{01}\left(x,x\right)\right], \end{array}$$

in Equation (101) with the symmetry $\Delta_{10}=\Delta_{-10}$ and $\Delta_{01}=\Delta_{0-1}$. It appears in taking the time derivative of $J_{1}$ (given by Equation (21)) in Equation (20). Here $\Delta_{10}\left(x,x\right)$ and $\Delta_{01}\left(x,x\right)$ can be rewritten by,

(A1) $$\begin{array}{lll} \Delta_{10}\left(x,x\right) & = & -\frac{ed_{e}}{i}\int_{w}\Delta_{g,11}\left(x,w\right)E_{1}\left(w\right)\Delta_{00}\left(w,x\right), \end{array}$$

(A2) $$\begin{array}{lll} \Delta_{01}\left(x,x\right) & = & -\frac{ed_{e}}{i}\int_{w}\Delta_{00}\left(x,w\right)E_{1}\left(w\right)\Delta_{g,11}\left(w,x\right), \end{array}$$

where we have used Equations (31) and (34) by setting $E_{2}=0$. We rewrite second time derivatives of $\Delta_{10}\left(x,x\right)$ and $\Delta_{01}\left(x,x\right)$.

We shall rewrite Equation (30) without self-energy $\Sigma_{\alpha \alpha }$ as,

(A3) $$\begin{array}{lll} \left[i\frac{\partial }{\partial x^{0}}+\frac{\nabla_{i}^{2}}{2m}-\frac{1}{2I}\right]\Delta_{g,11}\left(x,w\right) & = & i\delta_{C}\left(x-w\right), \end{array}$$

(A4) $$\begin{array}{lll} \left[-i\frac{\partial }{\partial x^{0}}+\frac{\nabla_{i}^{2}}{2m}-\frac{1}{2I}\right]\Delta_{g,11}\left(w,x\right) & = & i\delta_{C}\left(w-x\right), \end{array}$$

where we have multiplied $\Delta_{g,11}$ from the right and left of Equation (30). By using the above equations and Equations (32) and (33) with Equations (A1) and (A2) and $\Delta_{0,00}^{-1}\left(x,y\right)=\left(i\frac{\partial }{\partial x^{0}}+\frac{\nabla_{i}^{2}}{2m}\right)\delta_{C}\left(x-y\right)$, we can show

(A5) $$\begin{array}{lll} \frac{\partial }{\partial x^{0}}\Delta_{10}\left(x,x\right) & = & ed_{e}[\left[\left(-\frac{\nabla_{i}^{2}}{2m}+\frac{1}{2I}\right)\Delta_{g,11}+i\delta_{C}\right]E_{1}\Delta_{00}\\ & & +\Delta_{g,11}E_{1}\frac{\nabla_{i}^{2}}{2m}\Delta_{00}+2\Delta_{g,11}ed_{e}E_{1}\Delta_{01}E_{1}-\Delta_{g,11}E_{1}i\delta_{C}]\\ & = & ed_{e}\left[\left(\frac{1}{2I}\Delta_{g,11}+i\delta_{C}\right)E_{1}\Delta_{00}+2\Delta_{g,11}ed_{e}E_{1}\Delta_{01}E_{1}-\Delta_{g,11}E_{1}i\delta_{C}\right], \end{array}$$

(A6) $$\begin{array}{lll} \frac{\partial }{\partial x^{0}}\Delta_{01}\left(x,x\right) & = & ed_{e}\left[\left(-2ed_{e}E_{1}\Delta_{10}+i\delta_{C}\right)E_{1}\Delta_{g,11}+\Delta_{00}E_{1}\left(-\frac{1}{2I}\Delta_{g,11}i\delta_{C}\right)\right], \end{array}$$

where $\delta_{C}$ represents the delta function in the closed-time path. Here the terms proportional to $\nabla_{i}^{2}$ are cancelled in spatially homogeneous systems. By use of the above two equations, we can show

(A7) $$\begin{array}{l} \frac{\partial }{\partial x^{0}}\left(\Delta_{10}+\Delta_{01}\right)=\frac{1}{2iI}\left(\Delta_{10}-\Delta_{01}\right), \end{array}$$

and,

(A8) $$\begin{array}{lll} \frac{\partial^{2}}{\partial {\left(x^{0}\right)}^{2}}\left(\Delta_{10}+\Delta_{01}\right) & = & \frac{ed_{e}}{2iI}[\left(\frac{\Delta_{g,11}}{2I}+i\delta_{C}\right)E_{1}\Delta_{00}+2\Delta_{g,11}ed_{e}E_{1}\Delta_{01}E_{1}-\Delta_{g,11}E_{1}i\delta_{C}\\ & & -\left(-2ed_{e}E_{1}\Delta_{10}+i\delta_{C}\right)E_{1}\Delta_{g,11}-\Delta_{00}E_{1}\left(-\frac{1}{2I}\Delta_{g,11}-i\delta_{C}\right)]\\ & = & \frac{ed_{e}}{2iI}[2iE_{1}\left(\Delta_{00}-\Delta_{g,11}\right)+\frac{1}{2I}\left(\Delta_{g,11}E_{1}\Delta_{00}+\Delta_{00}E_{1}\Delta_{g,11}\right)\\ & & +2ed_{e}\left(\Delta_{g,11}E_{1}\Delta_{01}E_{1}+E_{1}\Delta_{10}E_{1}\Delta_{g,11}\right)]. \end{array}$$

Since we can rewrite Equations (35) or (36) by multiplying $i\Delta_{g,11}$ as,

(A9) $$\begin{array}{lll} i\Delta_{g,11}-i\Delta_{11} & = & ed_{e}\Delta_{g,11}E_{1}\Delta_{01}\\ & = & ed_{e}\Delta_{10}E_{1}\Delta_{g,11}, \end{array}$$

we arrive at,

(A10) $$\begin{array}{lll} \frac{\partial^{2}}{\partial {\left(x^{0}\right)}^{2}}\left(\Delta_{10}+\Delta_{01}\right) & = & -\frac{1}{4I^{2}}\left(\Delta_{10}+\Delta_{01}\right)+\frac{ed_{e}E_{1}}{I}\left(\Delta_{00}-2\Delta_{11}+\Delta_{g,11}\right), \end{array}$$

where we have used Equations (A1) and (A2).

Finally by rewriting the statistical parts (subscript ‘*F*’) of $\Delta_{10}+\Delta_{01}$ with Equations (A1) and (A2), and using $E_{1}\left(w\right)=E_{1}\left(x\right)+\left(w^{0}-x^{0}\right)\partial_{0}E_{1}\left(x\right)$ in,

$$\begin{array}{l} {\left[\int dw\left[\Delta_{g,11}\left(x,w\right)E_{1}\left(w\right)\Delta_{00}\left(w,x\right)+\Delta_{00}\left(x,w\right)E_{1}\left(w\right)\Delta_{g,11}\left(w,x\right)\right]\right]}_{F}, \end{array}$$

and the relation in the first order in the gradient expansion,

(A11) $$\begin{array}{lll} {\left[\int dw\Delta_{g,11}\left(x,w\right)\Delta_{00}\left(w,x\right)\right]}_{F} & = & \int_{p}(\frac{\Delta_{g,11,R}\left(x,p\right)}{i}F_{00}\left(x,p\right)+\Delta_{g,11,F}\frac{\Delta_{00,A}}{i}\\ & & +\frac{i}{2}\left\{\frac{\Delta_{g,11,R}\left(x,p\right)}{i},F_{00}\left(x,p\right)\right\}+\frac{i}{2}\left\{\Delta_{g,11,F},\frac{\Delta_{00,A}}{i}\right\}), \end{array}$$

with the advanced (subscript ‘*A*’) Δ00,A=i(Δ0011−Δ0021)=ReΔ00,R−ρ002 and the retarded Δ00,R=i(Δ0011−Δ0012)=ReΔ00,R+ρ002, we can derive the third, fourth and fifth terms on the right-hand side in Equation (121).
